# Gut microbiome modifications over time when removing in-feed antibiotics from the prophylaxis of post-weaning diarrhea in piglets

**DOI:** 10.1371/journal.pone.0262199

**Published:** 2022-03-07

**Authors:** Paola Cremonesi, Filippo Biscarini, Bianca Castiglioni, Carlo Angelo Sgoifo, Riccardo Compiani, Paolo Moroni

**Affiliations:** 1 National Research Council, Institute of Biology and Biotechnology in Agriculture (CNR-IBBA), Milan, Italy; 2 Department of Veterinary Medicine (Unimi-Medvet), University of Milan, Milan-Lodi, Italy; 3 Department of Veterinary Science for Health, Animal Production and Food Safety (Unimi-Vespa), University of Milan, Milan-Lodi, Italy; INRAE, FRANCE

## Abstract

Post-weaning diarrhea (PWD) in pigs has mainly an infectious basis and control strategies are centred on antibiotics added to the diet. Given concerns on the spread of multi-resistant bacteria, it is necessary to develop alternative prophylactic approaches to control PWD in piglets. The most promising alternative strategies are based on substances that act indirectly on the bacteria by stimulating the immune system or by improving gut health. The aim of this study was to evaluate the effect on the gut microbiota of feed supplemented with a mixture of essential oils (garlic and oregano) in weaning piglets, compared to traditional PWD management (in-feed antibiotics) and to a control group without any diet supplementation. The study involved 197 piglets from 18 litters in a single farm. The piglets were followed from birth to day 58 of age and were weaned at day 26. During the experimental period, the animals were monitored for weight and growth, average daily gain, morbidity and mortality. For the metataxonomics analysis, rectal samples were collected from 17 piglets from the three experimental groups at 4 different time-points (days 1, 12, 26 and 58). Results revealed that the gut microbiota in pre- and post-weaning piglets was dominated by the phyla *Firmicutes* (51%), *Bacteroidetes* (25%) and *Proteobacteria* (16%), which together make up for over 90% of the entire piglet core gut microbiota. The core microbiota comprised 10 taxa before weaning and 43 taxa after weaning, with 7 taxa overlapping between timepoints: two of them (*Prevotella 9*, p-value = 0.00095; *Solobacterium* p-value = 0.00821) were significantly more abundant after weaning. All alpha diversity indexes were significantly different between pre- and post-weaning, while only Shannon and Simpson diversity and equitability were significantly different between treatments. Based on the matrix of Bray-Curtis dissimilarities, samples showed clear clustering per timepoint (before and after weaning, p-value < 0.001) and between treatments by timepoint (p-value = 0.0086). The oil-diet group showed a consistently higher F:B ratio at all timepoints. These results show that the pig gut microbiota changes significantly with weaning, and suggest that the use of essential oils as feed supplementation to control PWD does not seem to alter sgnificantly the microbiota nor the growth parameters of piglets, however modifications of specific taxa may occur.

## Introduction

Post-weaning diarrhea (PWD) is among the most menacing threats to the pig industry worldwide and causes relevant economic losses due to early mortality, weight loss, slow growth and treatment costs [1]. PWD is a multifactorial disease with a predominant infectious basis which includes Gram-negative bacteria like *Escherichia coli* (especially enterotoxigenic strains, ETEC), Campylobacter spp. and Salmonella spp., rotavirus, in conjunction with dietary changes, abiotic stress and mismanagement [2]. The prevalence of PWD has been reported to be around 24% in Australia [3] and 30% in the US [4], with mortality that may reach 20–30% among affected piglets [2], thus standing out as a major health and management problem in pig-weaning operations.

Traditional strategies to control PWD are centered on antibiotics added to the diet for weaning piglets [5]: colistin (polymixin E) is the most widely used antibiotic against PWD in pigs, alone or combined with other antimicrobials like amoxicillin, given its efficacy and low cost [2]. However, the emergence of colistin-resistant strains in pigs affected by PWD [6, 7] is a serious public health concern, since colistin is a last-line therapeutic to treat multidrug resistant Gram-negative bacterial infections in humans [8–10]. This concern, together with resistance to other antimicrobials used for the treatment of PWD [11, 12], and the general apprehension on antibiotic resistance in livestock and the potential spread to humans, prompted actions to restrict the use of antibiotics in food-producing animals [13], in pigs specifically [5]. In addition, several countries (Sweden in 1986, Denmark in 1998, the EU in 2006) have introduced bans on the prophylactic use of in-feed antibiotics, precisely to tackle the issue of antibiotic resistance in humans (e.g. Regulation 1831/2003/EC on additives for use in animal nutrition). Therefore, ongoing research is investigating the adoption of alternative strategies to control PWD in piglets, like trace minerals, probiotics, prebiotics, organic acids, bacteriophages, oral vaccines (specifically towards F4 and F18-positive enterotoxigenic *E. coli* strains) [1, 2, 5, 14, 15]. Essential oils are mixtures of volatile organic compounds from diverse plant species that are evaluated as an additional non-antimicrobial means to restore intestinal balance and manage the weaning transition. Some of the essential oils evaluated so far in pigs include carvacrol, thymol and cinnamaldehyde, in different proportions [16].

Most of these alternative strategies to control PWD act indirectly on pathogenic bacteria by stimulating the immune system, possibly through the gut-brain axis [17], or by modulating the gut microbiome. Previous analysis of the pig gut microbiome already showed that withholding prophylactic antibiotics from the diet reshapes its composition and function [18]. It is therefore relevant to evaluate the effect of feed additives alternative to antibiotics on the gut microbiome of post-weaning piglets.

The aim of the present study was to compare traditional PWD control strategy based on the prophylactic use of antibiotics and antiparasitics (traditional diet: toltrazuril, ceftiofur, amoxicillin, enrofloxacin) in the diet of piglets to an alternative treatment based on feed supplemented with a mixture of essential oils (oil diet: garlic and oregano), and to a control group with just the normal diet and no supplementations (white diet: no antibiotics, no oil), for their effect on the gut microbiome.

## Results

### Growth performance and health conditions

Seventeen (N = 17) piglets of the same Danish commercial hybrid were selected from one herd for the 16S rRNA-gene sequencing of rectal samples collected at four time points (T0, T1: before weaning; T2, T3: from weaning onward). The 17 piglets belonged to three experimental groups: white diet (n = 5; normal diet, no antibiotics nor essential oils added to the diet), traditional diet (n = 6; antibiotics added to the diet), oil diet (n = 6; essential oils added to the diet), and were chosen out of a larger group subjected to the same treatments (N = 197: white = 57, traditional = 68, oil = 72).

Growth and health status were evaluated on all components of the three groups (Table 1): the body weight (BW) at birth was the same, and progressively diverged at weaning (p-value = 0.0698) and at the end of the experiment (p-value = 0.0012). The average daily gain (ADG) was significantly different between groups before (p-value = 0.0204) and after (p-value = 0.0228) weaning. A lower fraction of the piglets that did not receive antibiotic prophylaxis in the diet (white and oil groups) survived through weaning (87.7% and 83.3% in the white and oil groups, compared to 92.7% in the traditional group), although this difference was not significant (p-value = 0.2450). After weaning, mortality was not dissimilar between groups (traditional: 1.47%; white: 1.75%; oil: 1.39%). In the sequenced subset, piglets from the white group had a lower BW at weaning (5.7 kg compared to 7.4 kg and 7.3 kg in the traditional and oil groups), a significant between-group difference in ADG before weaning was found, but no significant between-group differences were found for BW at the end of the experiment and for ADG after weaning.

**Table 1:** Mortality, growth performance and individual therapeutic treatments in piglets from the three groups (white diet, traditional diet, oil diet).

sample	group	N	BW d1	BW d26	BW end	ADG d1–26	ADG d26-end	ADG overall	mortality pre	mortality post
all	white	57	1.279	6.907	19.992	0.215	0.409	0.352	0.123	0.018
	traditional	68	1.248	7.444	20.685	0.240	0.412	0.361	0.074	0.015
	oil	72	1.240	6.944	20.699	0.221	0.428	0.367	0.167	0.014
	p-value		0.793	0.070	0.001	0.020	0.023		0.245	0.985
sequenced	white	5	1.310	5.720	20.342	0.170	0.457	0.372	0.000	0.000
	traditional	6	1.342	7.392	20.966	0.233	0.424	0.368	0.000	0.000
	oil	6	1.350	7.333	21.019	0.230	0.428	0.369	0.000	0.000
	p-value		0.957	0.012	0.208	0.024	0.236			

BW: body weight; ADG: average daily gain; mortality pre and post refer to mortality before and after weaning. p-values from ANOVA.

### Sequencing metrics

From 68 rectal swab samples the bacterial 16S rRNA-gene sequencing of the V3-V4 regions produced a total of 7,365,257 reads with an average of 108,312 reads per sample. After quality filtering, 1,543,679 sequences were removed, leaving 5,821,578 sequences for subsequent analyses (78.25% average retention rate, maximum 82.4%, minimum 58.6%). The average number of sequences per treatment and time-point is reported in S1 Table: this varies from a minimum of 79,070 (±46601) in the “oil” experimental group to a maximum of 97,439 (±82492) in the “white” group.

The initial number of OTUs identified was 14,291; after removing OTUs with < 10 counts in 2 samples or less, 4066 distinct OTUs were left. To check whether sequencing depth and sample size were adequate to characterize the composition of the pig gut microbiota, sequence-based and sample-based rarefaction curves were generated from the OTU table before filtering. The observed number of OTUs detected was plotted as a function of the number of reads (up to 45,000) in each sample and of the number of samples (S1 Fig). Both curves tend to plateau asymptotically towards a maximum, indicating that sequencing depth and the number of samples were adequate to characterize the gut piglets’ microbiota in the present study. Deeper sequencing or the addition of any other samples would likely not increase significantly the number of new OTUs discovered.

### Core gut microbiome pre- and post-weaning

The 16S rRNA-gene sequencing results from all samples were used to characterize the core pig rectal microbiome. OTUs were grouped taxonomically from the phylum to genus level (phylum, class, order, family, genus). The 4066 OTUs with more than 10 counts in at least two samples clustered into 20 distinct phyla, 35 classes, 56 orders, 94 families and 318 genera. Considering OTUs shared by at least 90% of the samples, the pig core rectal microbiota comprised predominantly *Firmicutes*, *Proteobacteria* and *Bacteroidetes* with *Actinobacteria* and *Fusobacteria* as minority phyla, which accounted for over 90% of the entire piglets core gut microbiota (Table 2). The core microbiota comprised 10 taxa before weaning (T0 and T1) and 43 taxa after weaning (T2 and T3), with 7 taxa overlapping between timepoints: two of the latter were significantly different in the core gut microbiota of piglets before and after weaning, the genera *Prevotella 9* (p-value = 0.00095) and *Solobacterium* (p-value = 0.00821).

**Table 2:** Distinct OTUs included in the pig core gut microbiome (> 90% of the samples) before (pre) and after (post) weaning.

time	phylum	class	order	family	genus	avg counts
pre	Bacteroidetes	Bacteroidia	Bacteroidales	Bacteroidaceae	Bacteroides	1701.22
	Bacteroidetes	Bacteroidia	Bacteroidales	Prevotellaceae	Prevotella 9	379.38
	Firmicutes	Bacilli	Lactobacillales	Lactobacillaceae	Lactobacillus	235.67
	Firmicutes	Clostridia	Clostridiales	Clostridiaceae 1	Clostridium sensu stricto 1	12.26
	Firmicutes	Erysipelotrichia	Erysipelotrichales	Erysipelotrichaceae	Solobacterium	279.41
	Firmicutes	Negativicutes	Selenomonadales	Acidaminococcaceae	Phascolarctobacterium	716.12
	Firmicutes	Negativicutes	Selenomonadales	Veillonellaceae	Veillonella	127.03
	Fusobacteria	Fusobacteriia	Fusobacteriales	CFT112H7	uncultured bacterium	1607.82
	Fusobacteria	Fusobacteriia	Fusobacteriales	Fusobacteriaceae	Fusobacterium	2221.41
	Proteobacteria	Gammaproteobacteria	Enterobacteriales	Enterobacteriaceae	Escherichia-Shigella	6867.47
post	Actinobacteria	Coriobacteriia	Coriobacteriales	Coriobacteriaceae	Collinsella	71.94
	Bacteroidetes	Bacteroidia	Bacteroidales	Bacteroidaceae	Bacteroides	394.49
	Bacteroidetes	Bacteroidia	Bacteroidales	Bacteroidales S24–7 group	uncultured bacterium	837.35
	Bacteroidetes	Bacteroidia	Bacteroidales	Prevotellaceae	Alloprevotella	345.68
	Bacteroidetes	Bacteroidia	Bacteroidales	Prevotellaceae	Prevotella 1	637.91
	Bacteroidetes	Bacteroidia	Bacteroidales	Prevotellaceae	Prevotella 2	559.63
	Bacteroidetes	Bacteroidia	Bacteroidales	Prevotellaceae	Prevotella 7	2034.15
	Bacteroidetes	Bacteroidia	Bacteroidales	Prevotellaceae	Prevotella 9	1844.45
	Bacteroidetes	Bacteroidia	Bacteroidales	Prevotellaceae	Prevotellaceae NK3B31 group	451.12
	Bacteroidetes	Bacteroidia	Bacteroidales	Prevotellaceae	Prevotellaceae UCG-001	484.29
	Bacteroidetes	Bacteroidia	Bacteroidales	Prevotellaceae	uncultured	778.53
	Bacteroidetes	Bacteroidia	Bacteroidales	Rikenellaceae	Rikenellaceae RC9 gut group	639.62
	Bacteroidetes	Bacteroidia	Bacteroidales	uncultured	uncultured bacterium	136.62
	Firmicutes	Bacilli	Lactobacillales	Lactobacillaceae	Lactobacillus	327.90
	Firmicutes	Clostridia	Clostridiales	Lachnospiraceae	[Ruminococcus] gauvreauii group	59.41
	Firmicutes	Clostridia	Clostridiales	Lachnospiraceae	Blautia	74.71
	Firmicutes	Clostridia	Clostridiales	Lachnospiraceae	Coprococcus 3	31.41
	Firmicutes	Clostridia	Clostridiales	Lachnospiraceae	Dorea	128.53
	Firmicutes	Clostridia	Clostridiales	Lachnospiraceae	Fusicatenibacter	98.26
	Firmicutes	Clostridia	Clostridiales	Lachnospiraceae	Lachnoclostridium	63.80
	Firmicutes	Clostridia	Clostridiales	Lachnospiraceae	Lachnospiraceae NK4A136 group	109.21
	Firmicutes	Clostridia	Clostridiales	Lachnospiraceae	Lachnospiraceae UCG-004	670.65
	Firmicutes	Clostridia	Clostridiales	Lachnospiraceae	Lachnospiraceae UCG-008	9.71
	Firmicutes	Clostridia	Clostridiales	Lachnospiraceae	Pseudobutyrivibrio	447.88
	Firmicutes	Clostridia	Clostridiales	Lachnospiraceae	Roseburia	734.65
	Firmicutes	Clostridia	Clostridiales	Lachnospiraceae	uncultured	21.03
	Firmicutes	Clostridia	Clostridiales	Ruminococcaceae	[Eubacterium] coprostanoligenes group	509.50
	Firmicutes	Clostridia	Clostridiales	Ruminococcaceae	Faecalibacterium	588.15
	Firmicutes	Clostridia	Clostridiales	Ruminococcaceae	Ruminiclostridium 9	184.18
	Firmicutes	Clostridia	Clostridiales	Ruminococcaceae	Ruminococcaceae UCG-002	532.85
	Firmicutes	Clostridia	Clostridiales	Ruminococcaceae	Ruminococcaceae UCG-005	112.04
	Firmicutes	Clostridia	Clostridiales	Ruminococcaceae	Ruminococcaceae UCG-014	89.06
	Firmicutes	Clostridia	Clostridiales	Ruminococcaceae	Subdoligranulum	147.72
	Firmicutes	Clostridia	Clostridiales	Ruminococcaceae	uncultured	18.94
	Firmicutes	Erysipelotrichia	Erysipelotrichales	Erysipelotrichaceae	Catenibacterium	81.85
	Firmicutes	Erysipelotrichia	Erysipelotrichales	Erysipelotrichaceae	Solobacterium	2971.06
	Firmicutes	Negativicutes	Selenomonadales	Acidaminococcaceae	Acidaminococcus	555.88
	Firmicutes	Negativicutes	Selenomonadales	Acidaminococcaceae	Phascolarctobacterium	719.92
	Firmicutes	Negativicutes	Selenomonadales	Veillonellaceae	Anaerovibrio	1776.03
	Firmicutes	Negativicutes	Selenomonadales	Veillonellaceae	Megasphaera	740.88
	Fusobacteria	Fusobacteriia	Fusobacteriales	CFT112H7	uncultured bacterium	362.94
	Proteobacteria	Epsilonproteobacteria	Campylobacterales	Helicobacteraceae	Helicobacter	597.76
	Proteobacteria	Gammaproteobacteria	Enterobacteriales	Enterobacteriaceae	Escherichia-Shigella	943.12

### Gut microbiome over time and treatments

**Fig 1:**
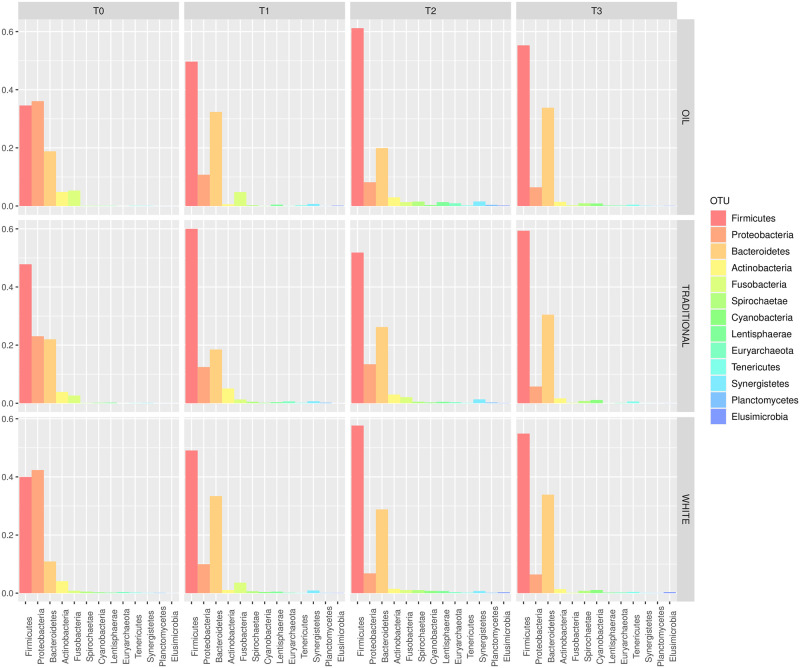
Phylum distribution. Relative abundance of phyla per treatment and timepoint in the gut microbiota of piglets.

**Fig 2:**
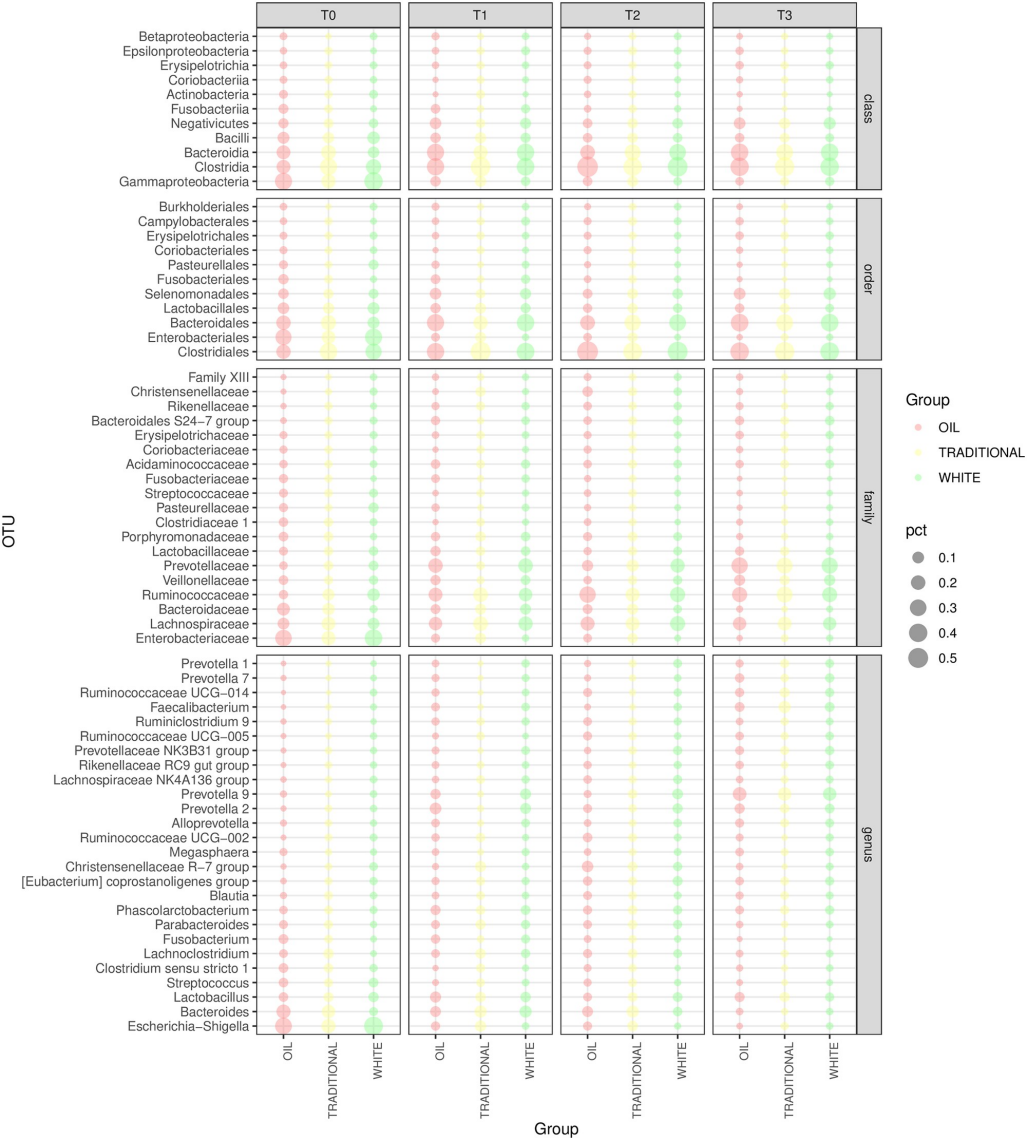
Relative distribution of taxonomic levels. Relative abundance of classes, orders, families and genera in the piglet gut microbiota between treatments (traditional diet, white diet, oil diet) and over time; only taxa with relative abundance > 0.001 are shown.

**Fig 3:**
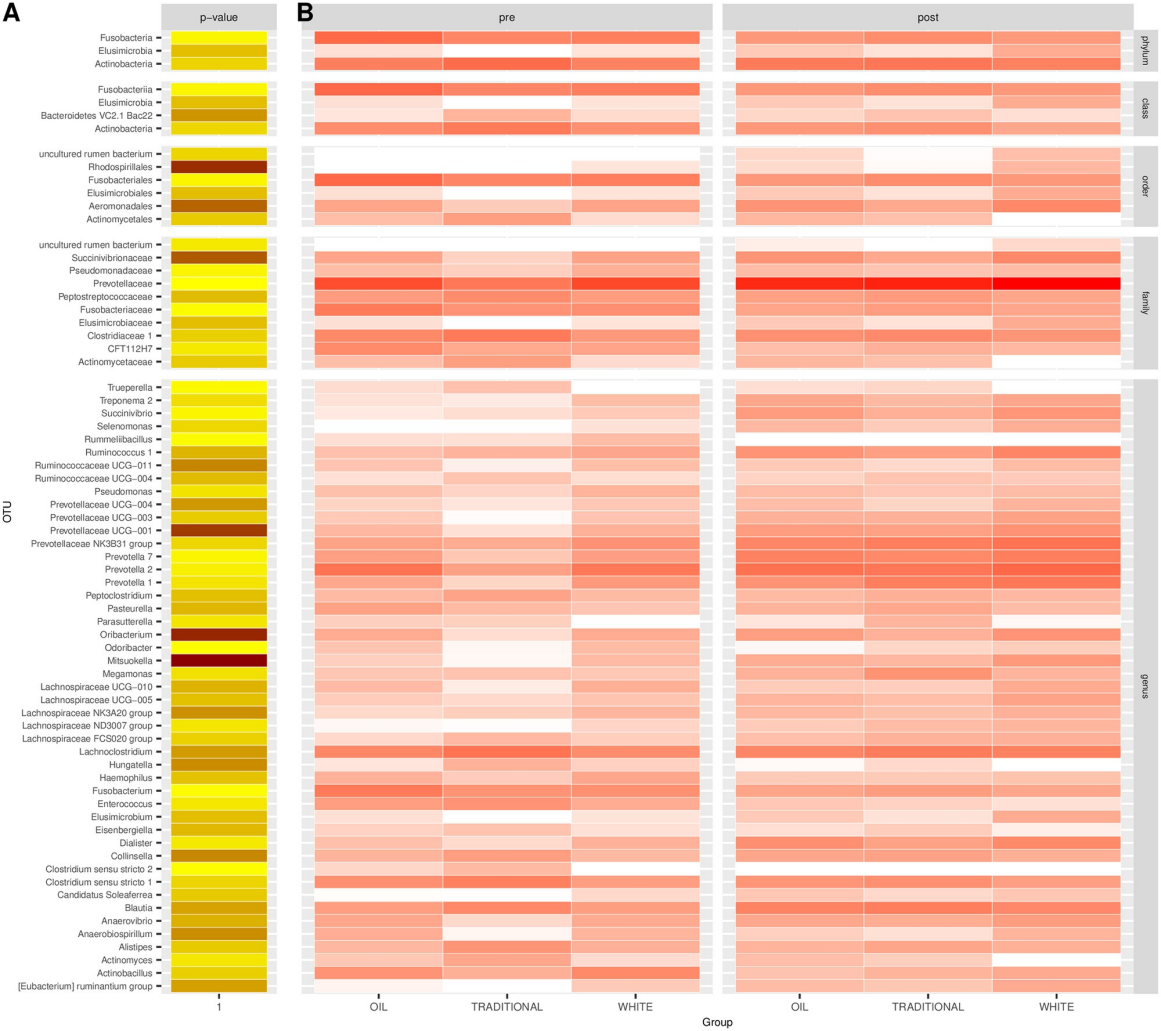
Significantly different OTUs. OTU significantly different between treatments from analysis of variance based on normalised counts: p-values (A) and pre- and post-weaning per-group counts (B). p-value <0.05 was used as cut-off. Darker colors indicate lower p-values (A) or higher counts (B). Full results are reported in S4 Table.

Fig 1 shows the relative abundance of phyla in the piglet gut microbiota across PWD prophylactic treatments (white diet, traditional diet, oil diet) and over time (from T0 to T3). Phyla with relative abundance ≤0.1% were not considered (full results in S2 Table). *Firmicutes* (40.8%) and *Proteobacteria* (33.8%) were found to be the most abundant phyla in the gut microbiota at T0, followed by *Bacteroidetes* (17.2%), *Actinobacteria* (4.3%) and *Fusobacteria* (2.9%). From T1 to T3, *Bacteroidetes* replaced *Proteobacteria* as the second-most abundant phylum (28.1%, 24.9% and 32.7% vs 11.1%, 9.5% and 6.2% at T1, T2 and T3 respectively). Fig 2 shows the relative abundances of classes, orders, families and genera across treatments and timepoints. OTU with relative abundance ≤0.25% were not considered (the complete list of OTU relative abundances between treatments and over time can be found in S3 Table). From the linear model of normalised OTU counts over treatments and timepoints (Eq 1), 70 OTU from different taxonomic levels were found to be significantly different between treatments (p-value < 0.05, Fig 3 and S4 Table).

### F:B ratio

**Fig 4:**
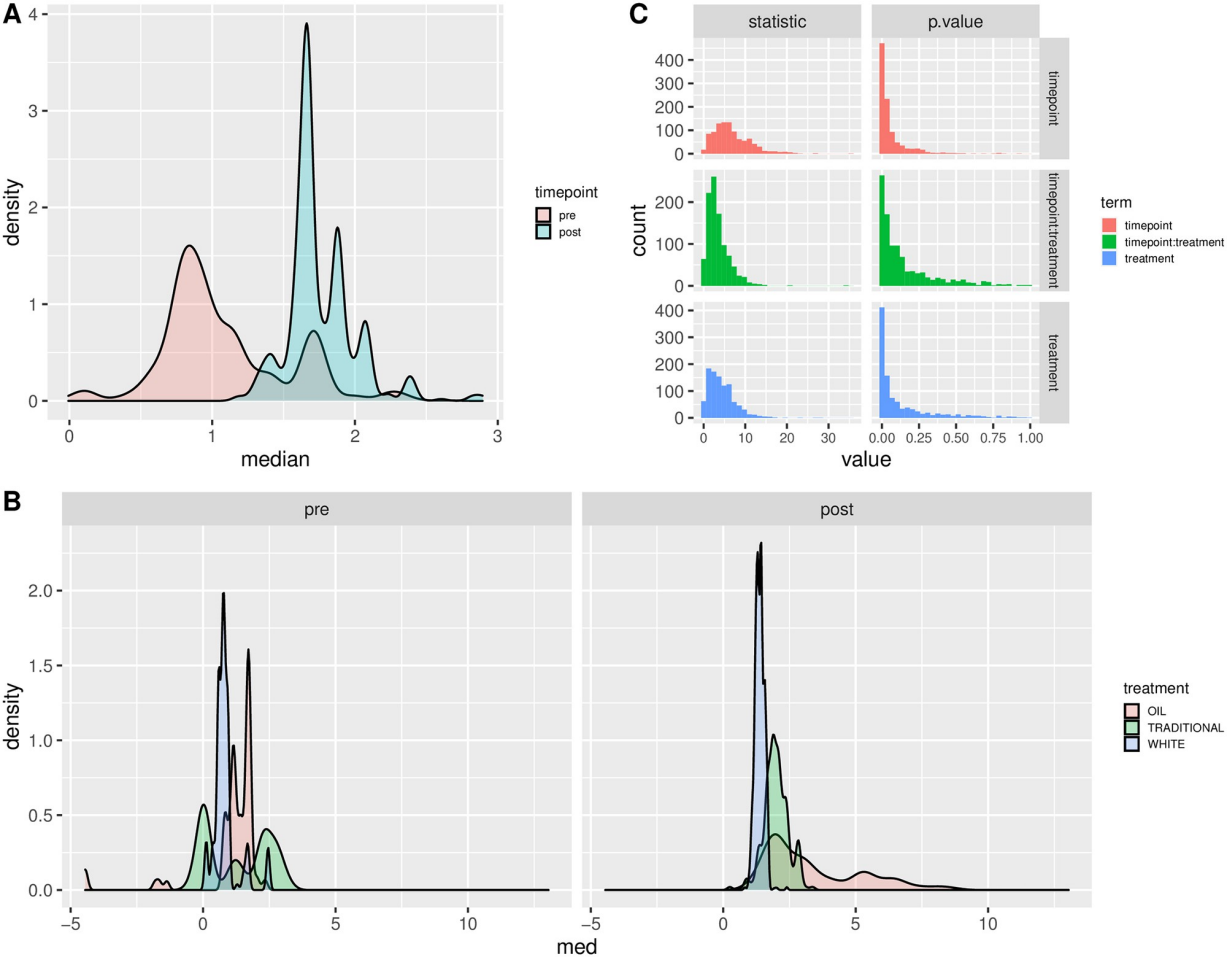
F:B ratio. F:B ratio before and after weaning (A) and between treatments at each time point (B). Results from 1000 bootstrapped replicates of the dataset. Results from bootstrapping regression (test statistic, p-values) are in pane C.

The F:B (Firmicutes to Bacteroidetes) ratio from normalised counts before and after weaning is reported in Table 3 for the three experimental groups. F:B ratio was higher after weaning and in the oil-diet group, followed by the traditional and white diet groups. From bootstrapping (1000 replicates), the interquartile range around median F:B ratios was obtained. A clear difference was observed in F:B before and after weaning; the differences between treatments within time point were smaller in magnitude, but still present (Fig 4A and 4B). These differences were moderately to full significant, both from the original data configuration and from bootstrapping (Table 4): between-treatment p-values were 0.0526 (original data) and 0.0294 (bootstrapping); p-values for the before vs after weaning F:B ratio were 0.0236 (original data) and 0.0165 (bootstrapping). The interaction between time point and treatment was, on the other hand, not significant, indicating that the relationship between treatments (F:B ratio highest in the oil group followed in order by the traditional and white groups) did not change before and after weaning. This is further illustrated by the distribution of bootstrapped p-values (Fig 4C), with a more pronounced right-skewness of the distributions for the treatment and timepoint terms, compared to their interaction.

**Table 3:** F:B ratio between treatments and timepoints. Q1_bstr and Q3_bstr are the first and third quartiles from the bootstrapping distribution of the F:B ratio (n = 1000).

treatment	pre-weaning			post-weaning		
	median_FB	Q1_bstr	Q3_bstr	median_FB	Q1_bstr	Q3_bstr
OIL	1.423	1.146	1.699	2.541	1.970	5.265
TRADITIONAL	1.156	0.064	2.248	1.985	1.666	2.235
WHITE	0.772	0.587	0.939	1.363	1.254	1.497

**Table 4:** Significance of F:B differences between treatments and timepoints, and their interaction. statistic_bstr and p-value_bstr are the median test statistic and p-value from the bootstrapping distribution of 1000 replicates of the linear model.

term	statistic	p-value	statistic_bstr	p-value_bstr
timepoint	5.4859	0.0237	5.8806	0.0194
treatment	3.1455	0.0526	3.6617	0.0336
timepoint:treatment	2.8425	0.0688	2.7692	0.0734

### Alpha and beta diversity

**Fig 5:**
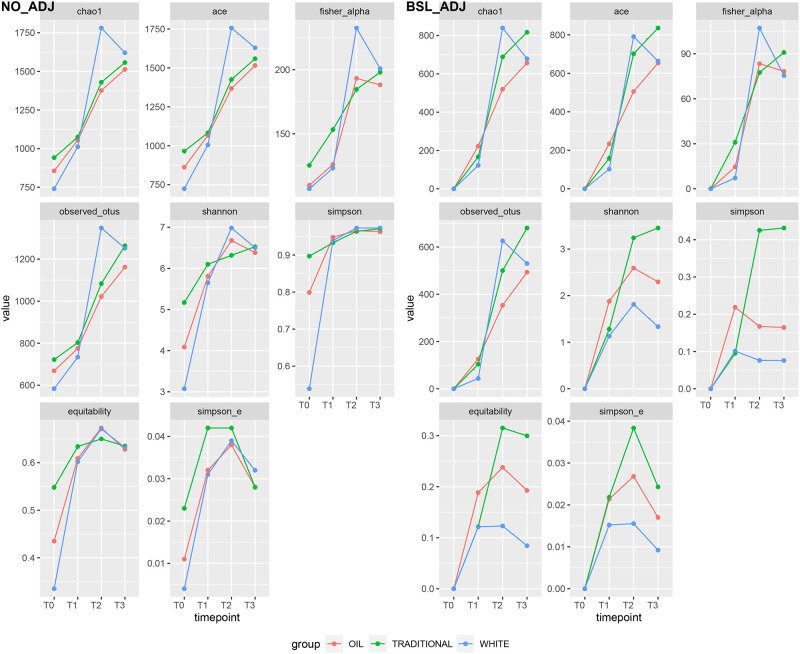
Alpha diversity. Average alpha diversity indices per group over timepoints. Non-adjusted (left) and baseline-adjusted (right) values.

**Fig 6:**
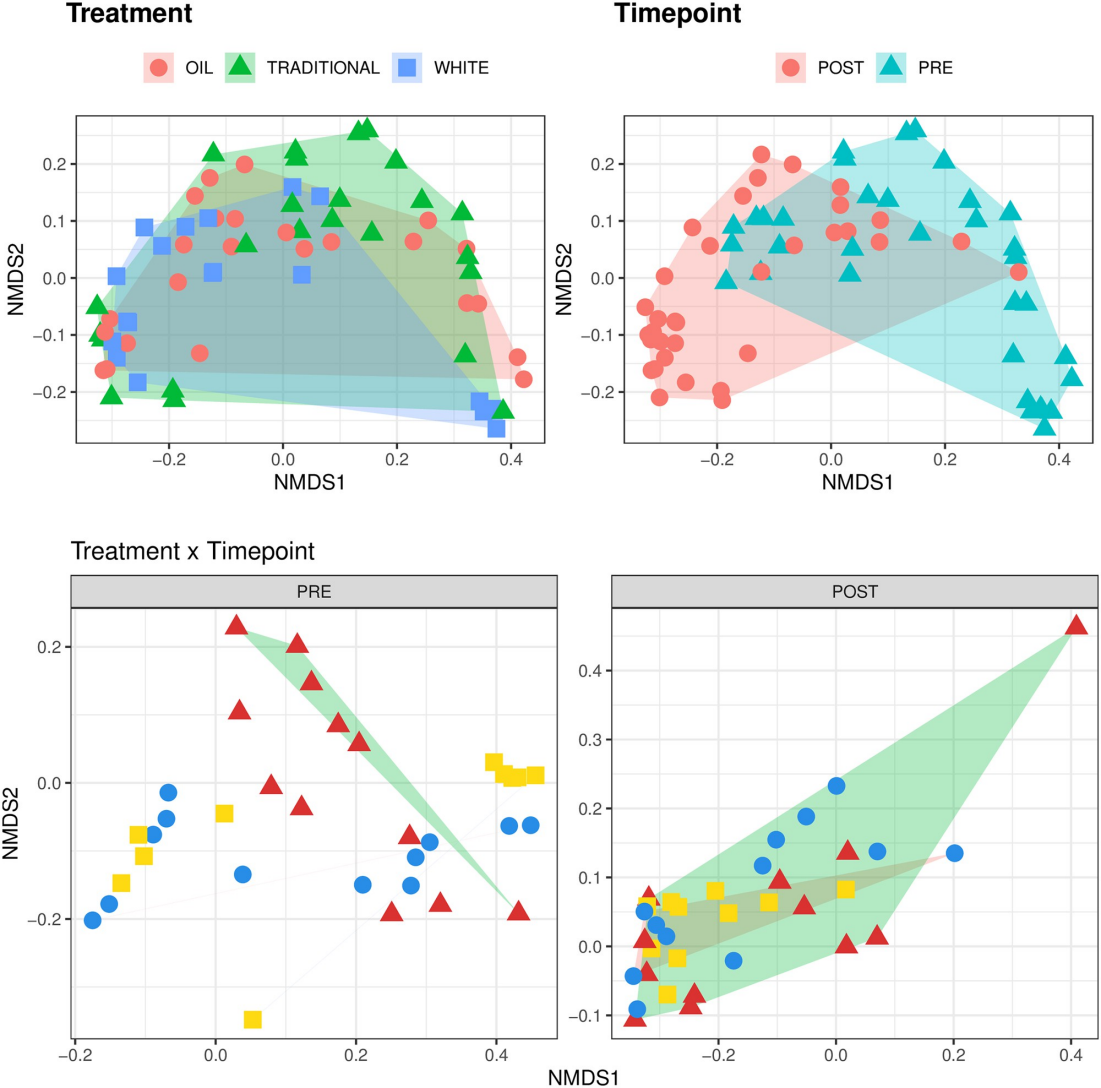
Beta diversity. First two dimensions from the (non-metric) multi-dimensional scaling (NMDS) of Bray-Curtis distances between samples. Samples were grouped by experimental units: by treatment on the upper left pane, by timepoint on the upper right pane, by treatment and timepoint in the lower pane. PERMANOVA among treatments p-value = 0.052, PERMANOVA among timepoints p-value = 0.001 (using 999 permutations), PERMANOVA among treatments by timepoint (pre- vs post-weaning) p-value = 0.0086.

Fig 5 shows the trends of alpha diversity indices over time for the three treatments: both raw index values (on the left) and index values adjusted by baseline (on the right, T0 used as reference, set to 0) are presented. From baseline-adjusted data, significant differences were found before and after weaning for all indices, and between treatments only for Shannon and Simpson diversity and for equitability (Table 5). Fig 6 shows the first two dimensions from the (non-metric) multi-dimensional scaling (NMDS) of Bray-Curtis distances between samples. Samples were grouped by experimental units: by treatment, by time point (pre- and post-weaning), and by treatment before and after weaning (day 26). From PERMANOVA (999 permutations), the between-treatment p-value was 0.052, the p-value for the difference before and after weaning was 0.001, and the treatment-by-timepoint interaction had a p-value = 0.0086.

**Table 5:** Alpha diversity indices and significance of differences between treatments, timepoints and their interaction.

indices	pre-weaning	post-weaning	pre-weaning			post-weaning			treatment	weaning
			wh.	oil	trad.	wh.	oil	trad.		
chao1	950.67	1537.03	876.37	955.26	1008.01	1700.61	1444.47	1493.26	0.3052217	1.15e-10
fisher_alpha	124.70	198.60	115.18	118.00	139.34	216.74	190.75	191.34	0.4259763	3.53e-10
observed_otus	717.44	1181.94	658.30	721.92	762.25	1300.00	1091.92	1173.58	0.5885714	5.95e-10
observed_species	717.44	1181.94	658.30	721.92	762.25	1300.00	1091.92	1173.58	0.5885714	5.95e-10
shannon	5.02	6.55	4.36	4.95	5.63	6.74	6.53	6.42	0.0019982	5.55e-09
simpson	0.85	0.97	0.74	0.87	0.91	0.97	0.96	0.97	0.0000131	3.21e-07
equitability	0.53	0.65	0.47	0.52	0.59	0.65	0.65	0.64	0.0012893	6.08e-07
simpson_e	0.02	0.03	0.02	0.02	0.03	0.04	0.03	0.04	0.4187639	3.51e-02
ACE	1477.18	2229.95	1285.03	1506.04	1608.43	2452.06	2124.21	2150.60	0.1367301	3.08e-10

## Discussion

Weaning is a complex and delicate physiological transition time in the life of pigs, when piglets are subject to a variety of biotic and abiotic stressors [19], especially related to sudden dietary, social, and environmental changes [16]. These stressors are causally associated with gastrointestinal infections, and this generally determines an increased use of antibiotics. Additionally, it is known that removing the prophylactic use of in-feed antibiotics leads to increased incidence of PWD, which results in slower growth, poorer productive performance and increased mortality [5, 20]. Non-antimicrobial alternatives, like essential oils, prebiotics or probiotics, zinc oxide, have been studied to evaluate the use of feed additives on the piglets’ intestinal microbiota at weaning [16]. In this paper, we monitored the gut microbiome of pigs in three treatment groups during the pre- and post-weaning period. Specifically, a comparison between the traditional PWD control strategy based on antibiotics and antiparasitics in the feed to an alternative treatment based on feed supplemented with a mixture of essential oils (garlic and oregano), and to a group with a basic diet and no supplementations (white) was performed. The relatively limited size of this experiment allowed to detect moderate-to-large between-group differences with sufficient statistical power (0.8 standard-deviation difference with 80% power at 0.05 significance), which was considered an acceptable trade-off between power and the number of animals involved. A larger-sized experiment would be needed to capture more subtle differences between treatments. Some relevant aspects of this study are discussed below.

### Core gut microbiome in the pre- and post-weaning phase

The core gut microbiome in the pre- and post-weaning phase was defined as OTU shared by ≥ 90% of the samples. As previously reported in pigs [21–23], during the pre-weaning period the most abundant taxa were the phyla *Firmicutes*, *Proteobacteria*, *Bacteroidetes* and *Fusobacteria*; the families *Enterobacteriaceae*, *Bacteroidaceae*, *Acidaminococcaceae*, *Lactobacillaceae* and *Fusobacteriaceae* accounted for over 80% of the entire gut microbiota. After weaning, *Firmicutes*, *Proteobacteria* and *Bacteroidetes* remained the predominant phyla with 7 taxa, the genera *Bacteroides*, *Prevotella*, *Lactobacillus*, *Solobacterium*, *Phascolarctobacterium*, *Escherichia-Shigella*, and the *Fusobacteriales* CFT112H7 group, overlapping between the two periods. Significant differences were found for *Prevotella 9* and *Solobacterium*. The increment in the genus *Prevotella* after weaning has been reported to be linked to the fermentation of plant-derived non-starch polysaccharides to short-chain fatty acids [19]. The same increase was observed for *Solobacterium* spp. indicating that different types of fiber could selectively regulate the intestinal bacteria [24]. Short-chain fatty acids have been reported to help maintain the gut homeostasis in pigs, thereby contributing to the prevention of PWD [25]. The relative abundance of *Lactobacillus* spp., recognized as a carbohydrate-utilizing bacterium, showed an increment from pre- to post-weaned piglets, suggesting that it might play a role in the degradation of complex carbohydrates [19]. Conversely, as previously reported [16, 19], the genus *Bacteroides* -that uses oligosaccharides from milk as sources of carbon- was more abundant in suckling piglets. The phylum *Proteobacteria* includes Gram-negative pathogenic bacteria such as the genera *Escherichia*, *Salmonella*, *Campylobacter*, *Helicobacter*, *Vibrio* and *Shigella*, usually considered as potential indicators of gut dysbiosis. Their decrement between pre- and post-weaning period, could be a sign of lower intestinal disorders.

### F:B ratio

In the present study, an increase of the F:B ratio in piglets from pre- to post-weaning was observed. The F:B ratio was higher in the oil-diet group, followed by the traditional (in-feed antibiotics) and white diet groups, both before and after weaning. From model 2, time differences (pre vs post weaning) were clearly significant, while treatment differences were barely significant. The bootstrapping resampling approach confirmed that the observed differences in F:B ratio were significant along both dimensions. The interaction between timepoints and treatments, on the other hand, was not significant in both approaches (linear model and bootstrapping), clearly indicating that the ordering of F:B ratio values (oil → antibiotics → white) was maintained unchanged before and after weaning. This is nicely illustrated also by the distribution of p-values for the interaction effect from bootstrapping (Fig 4C), which is markedly less right skewed compared to the p-value distributions for the timepoint and treatment effects, indicating that p-values from resampled copies of the data are less compressed towards zero (small values).

The F:B ratio has been reported to increase with age in the gut microbiomes of humans [26] and experimental mice models [27]; also in pigs, the F:B ratio was previously found to increase in the transition from piglets (1 month old) to adult pigs (2–3 months old) [28], which seems to suggest a pattern in omnivorous monogastric species. Additionally, the F:B ratio in the gut microbiota is known to play a role in adipogenesis: Jami et al. [29] observed a strong positive correlation between this ratio and milk-fat yield in dairy cows. In studies on obesity in mice and humans, it has also been related to higher blood and tissue fat [30, 31]. Also in pigs, the increase of this parameter is associated with an increment in the energy harvest and fat deposition [32] which, in the present study would translate to higher fat deposition after weaning (fattening phase) and in treated (oil and antibiotics) versus untreated (white) animals. This may relate to the long-standing observation of reduced growth in untreated pigs [33]. Lastly, the increase of Firmicutes with simultaneous reduction of Bacteroidetes in the gut microbiota may lead to dysbiosis (disruption of the bacterial balance), which in turn can promote intestinal colonization by pathogenic species such as *Campylobacter* spp. and *E. coli* ETEC [34].

### The pig gut microbiome in relation to dietary treatments

Essential oils are a mixture of volatile organic compounds with antimicrobial, antioxidant and antiviral properties, obtained from different plants [16]. Based on this premise, an alternative dietary prophylactic treatment based on a mixture of oregano (*Origanum vulgare* L.) and garlic (*Allium sativum*) was compared with the traditional PWD control strategy based on in-feed antibiotics and with a “white” diet without prophylactic supplementations, to evaluate the effects on the piglets’ gut microbiome during suckling (pre-) and post-weaning periods. As reported in previous studies [35–37], the use of essential oils appeared to improve both growth performance, intestinal function and antioxidant status, not only in pigs but also in other animal species (e.g. turkey [38]).

Across treatments the most abundant phyla were *Firmicutes*, *Bacteroidetes* and *Proteobacteria* (in this order), while there was a lower presence of *Actinobacteria* and *Fusobacteria*. This is in line with previous studies on gut bacterial diversity in piglets during the weaning transition period in association with health and nutrition [19, 21, 22, 39]. To reduce confounding from individual variability at the first sampling time (T0), alpha diversity indices were adjusted for baseline effect by removing the average values at day-1 (Fig 5, right pane): the white and oil diet groups appear to have lower adjusted diversity (Shannon, Simpson, Equitability and Simpson_E indices for the white-diet group, Chao1, ACE, Fisher’s alpha, observed n. of OTUs for the oil-diet group, respectively) compared to the traditional-diet group. Significant differences among treatments were found for the phyla *Actinobacteria* (p-value < 0.015), *Elusimicrobia* (p-value < 0.008) and *Fusobacteria* (p-value < 0.037), with 47 genera significantly different. *Lachnoclostridium*, *Prevotella 1*, *Prevotella 2*, *Prevotella 7* and *Prevotellaceae NK3B31 group* were the most abundant across treatments. *Prevotella* spp. is involved in the fermentation of non starch-polysaccharides [19], like *Lachnoclostridium*, a genus predicted to be involved in the degradation of dietary substrates [40]. *Blautia*, *Clostridium* sensu stricto, *Enterococcus*, *Alistipes* and *Megamonas* were more abundant with the traditional diet, while *Actinobacillus* and *Ruminococcus* with the white and oil diets. The genus *Blautia*, a short fatty acid-producing bacteria, was found to be correlated positively with growth performance and negatively with diarrhea incidence in piglets during the weaning period [41]. The genus *Ruminococcus* is known to be one of the essential taxa for metabolizing a wide range of complex oligosaccharides and polysaccharides for SCFAs production, facilitating the breakdown of proteins and carbohydrates in feed [32]. *Fusobacterium* was the most abundant genus in the oil group during the pre-weaning period. Under normal circumstances, strains from this genus are commensal and can produce butyrate from carbohydrates; however some *Fusobacterium* strains could be associated with severe gastrointestinal disease [22]. Previous studies [21, 37, 42] describing the use of essential oils, in particular oregano and garlic, as diet supplementation in weaning piglets, showed an increment of *Lactobacillus* followed by a decrease of *Enterobacteriaceae* relative abundance. *Lactobacillales* are recognized beneficial bacteria for the normal intestinal function and maintainance of corporal health [43], while the relative abundance of *Enterobacteriaceae* is positively correlated with a large number of intestinal inflammatory disorders [42]. In our study, these two taxa revealed the same trend but without significant differences among the three dietary treatments. As for *Megasphera*, *Butyrivibrio* and *Ruminococcus*, genera involved in producing short-chain fatty acids with a role in gut health [42], a significant increment of *Ruminococcus* spp. was detected with the oil and white diets.

### Gut microbiome during time

During the initial postnatal weeks, the gut microbiome in piglets is variable and prone to perturbations. In this phase, intestinal pathogens start to proliferate and can cause clinical disease [44]. The gut microbiota showed an evolution between pre- and post- weaning as indicated by the clustering over Bray-Curtis distances (Fig 6), which showed good separation (p-value < 0.001), and among treatments by timepoint (p-value = 0.0086). When considering only the time effect, 245 OTU showed significant difference in abundance before and after weaning (results not shown). The phyla *Bacteroidetes* and *Firmicutes* were dominant in the gut microbiota of weaning pigs, in agreement with Massacci et al. [45]. The genera *Bacteroides*, *Escherichia-Shigella*, *Faecalibacterium*, *Lachnoclostridium*, *Lactobacillus*, *Prevotella*, *Ruminococcaceae* and *Streptococcus* accounted for over 80% of the entire microbiota. Independently from treatment, there was a significant reduction from pre- to post-weaning of *Escherichia-Shigella* and other enteropathogenic bacteria that cause dysentery and diarrhea, as previously reported [39].

The genus *Bacteroides*, associated with breast milk and established as early colonizer of the pig hindgut, was significantly more abundant before weaning, confirming previous results [39, 46]. A shift from *Bacteroides* to *Prevotella* was observed during the weaning transition; *Prevotella* is generally linked to the fermentation of plant-derived non starch-polysaccharides to short chain fatty acids [19] and the genus *Prevotella 9* was the most abundant in post-weaning piglets microbiota, irrespective of treatments. *Streptococcaceae* and *Streptococcus* had been found to be negatively associated with inflammation in previous studies [47]. The higher relative abundance of *Streptococcaceae* in the traditional vs oil groups during pre- and post-weaning indicates that the intestinal health of weaned piglets was improved with the alternative PWD prophylactic diet.

Finally, *Enterococcus gallinarum* has been reported as a pathobiont associated with autoimmunity in humans and in mice models [48]. In this study, we found sizable amounts of *E. gallinarum* before weaning in the traditional (458 counts) and white (234 counts) diet groups, which virtually disappeared after weaning (6 and 19 counts for the traditional and white groups, respectively). Supplementation with the mixture of essential oils seems to drastically reduce the presence of *E. gallinarum* also before weaning (53 counts during the pre-weaning phase and 6 counts in post-weaning period).

## Materials and methods

### Ethics statement

This study was conducted on a single commercial farm in Corteolona (Pavia, Italy), in the framework of a collaborative relationship with the University of Milan. Trial animals were handled following the EU directive 86/609/ EEC concerning animal care and the guidelines of the Italian law on animal welfare for experimentation and ethics (Italian Health Ministry authorization n° OPBA_71_2019). Pigs were observed on a daily basis by one veterinarian and husbandry personnel under the supervision of the same veterinarian. Clinical signs, including temperature, were recorded. Sick pigs were given appropriate treatment and moved to the sick pen. Pigs unable to eat, drink or move or with no possibility of health status improvement were removed from the trial and humanely euthanized. Euthanasia was performed only by vets. No human endpoints at the end of the experiment were required by the study design since the trial consisted of monitoring the health and growth performance of piglets in a commercial farm under non-experimental conditions.

### Animals, treatments and sampling

The study involved 197 piglets from 18 litters in a single farm (Danish commercial hybrid). The piglets were followed from birth to day 58 of age and were weaned at day 26. During the experimental period, the animals were monitored for weight and growth (at day 1 (T0), day 12 (T1), day 26 (T2)—weaning, and day 58 (T3)—end of weaning), average daily gain, morbidity and mortality. In case of diarrhea, the analysis of biological feces, the necropsy on dead animals and the histological investigation were carried out to investigate the specific causes of diarrhea. The 197 piglets were randomly divided into 3 experimental groups on a per-litter basis (6 litters per treatment): i) basal diet without any treatments (white-diet group, n = 57), basal diet supplemented with antibiotics and antiparasitics (traditional-diet group, n = 68), and basal diet supplemented with a mixture of essential oils (oil-diet group, n = 72). All piglets received the same farm management and diet. The traditional group received individual treatments with toltrazuril and ceftiofur at day 1–7, amoxicillin at days 10 to 26 and enrofloxacin at days 27–58. This antibiotic protocol was based on the specific health history and management analysis of the commercial farm used for the experiment. The white and oil groups did not receive any antibiotic or antiparasitic treatments from birth to the end of weaning. The oil group received a supplementation with a mixture of essential oils (garlic and oregano, Coxsan^™^, 13% content of active ingredients) in the drinking water (500 g/1000 L). For the metataxonomics analysis, rectal samples were collected from 17 piglets (5 from the white, 6 from the traditional and 6 from the oil groups) at 4 different time-points: (i) day 1 before starting the treatments (T0); (ii) days 12 (T1) and 26 (T2); (iii) day 58 end of the weaning phase (T3). The piglets came from 4 litters (4 sows) and were randomly assigned to the three treatment groups. From day 1 to 10 piglets were kept in the delivery room; milk replacer and starch were fed to the piglets as support to natural milk. One group of piglets (oil) also received the natural additive (Coxsan™). Corn starch was used as a carrier for the natural additive administration, and it was used in each diet. From day 10 to day 58 (delivery room + weaning phase) the piglets were fed with solid feed plus the natural additive for the oil treatment group. After weaning, piglets were randomly assigned to post-weaning boxes, while obviously keeping the treatment to which they were assigned at the beginning of the experiment. The building was temperature-controlled during the study and feed and water were available ad libitum for all pigs. The diet composition is provided in Table 6.

**Table 6:** Sample size, diets and treatments (feed supplements) for the piglets selected for sequencing from the three experimental groups. From all pigs rectal samples were collected at day 1, 12, 26 and 58 for 16S rRNA-gene sequencing.

treatment group			piglets in the delivery-room		post-weaning
	N 16S	d1 (T0)	d2-d10 (T1)	d10-d26 (T2)	d26-d58 (T3)
Traditional diet	6	colostrum	milk replace + starch + toltrazuril and ceftiofur	solid feed + amoxicillin	solid feed + enrofloxacin
White diet	5	colostrum	milk replace + starch	solid feed	solid feed
Oil diet	6	colostrum	milk replace + starch and Coxsan^™^(500 g/1000 L)	solid feed + Coxsan^™^(500 g/1000 L)	solid feed + Coxsan^™^(500 g/1000 L)

### Sampling and sequencing

Rectal samples (rectal swabs) from the 17 randomly selected piglets were collected by rectal massage on day 1 (T0), 12 (T1), 26 (T2) and 58 (T3) and were stored at -80°C until DNA extraction. DNA was extracted from each sample using a QIAmp DNA Stool kit (Qiagen, Hilden, Germany), according to the manufacturer’s protocol with a minor modification. The rectal swabs were dissolved in 1 mL Buffer ASL and shaken at 1000 rpm (Mixing Block MB-102, CaRlibiotech S.r.l. Rome, Italy), continuously until the stool samples were homogenized. DNA quality and quantity were assessed using a NanoDrop ND-1000 spectrophotometer (NanoDrop Technologies, Wilmington, DE, USA). The isolated DNA was then stored at -20°C until use. Bacterial DNA was amplified using the primers described in literature [49] which target the V3-V4 hypervariable regions of the 16S rRNA gene. All PCR amplifications were performed in 25 μL volumes per sample. A total of 12.5 μL of KAPA HIFI Master Mix 2× (Kapa Biosystems, Inc., MA, USA) and 0.2 μL of each primer (100 μM) were added to 2 μL of genomic DNA (5 ng μL^−1^). Blank controls (no DNA template added to the reaction) were also performed. A first amplification step was performed in an Applied Biosystem 2700 thermal cycler (ThermoFisher Scientific). Samples were denatured at 95°C for 3 min, followed by 25 cycles with a denaturing step at 98°C for 30 s, annealing at 56°C for 1 min and extension at 72°C for 1 min, with a final extension at 72°C for 7 min. Amplicons were cleaned with Agencourt AMPure XP (Beckman, Coulter Brea, CA, USA) and libraries were prepared following the 16S Metagenomic Sequencing Library Preparation Protocol (Illumina, San Diego, CA, USA). The libraries obtained were quantified by Real Time PCR with KAPA Library Quantification Kits (Kapa Biosystems, Inc., MA, USA), pooled in equimolar proportion and sequenced in one MiSeq (Illumina) run with 2×250-base paired-end reads. The 16S rRNA gene sequences determined in this study were deposited in the NCBI Sequence Read Archive (SRA) database with the accession number PRJEB45296 (to be released).

### Bioinformatics processing

Demultiplexed paired-end reads from 16S rRNA-gene sequencing were first checked for quality using FastQC [50] for an initial assessment. Forward and reverse paired-end reads were joined into single reads using the C++ program SeqPrep [51]. After joining, reads were filtered for quality based on: i) maximum three consecutive low-quality base calls (Phred < 19) allowed; ii) fraction of consecutive high-quality base calls (Phred > 19) in a read over total read length ≥ 0.75; iii) no “N”-labeled bases (missing/uncalled) allowed. Reads that did not match all the above criteria were filtered out. All remaining reads were combined in a single FASTA file for the identification and quantification of OTUs (operational taxonomic units). Reads were aligned against the SILVA closed reference sequence collection release 123, with 97% cluster identity [52, 53], applying the CD-HIT clustering algorithm [54]. A pre-defined taxonomy map of reference sequences to taxonomies was then used for taxonomic identification along the main taxa ranks down to the genus level (domain, phylum, class, order, family, genus). By counting the abundance of each OTU, the OTU table was created and then grouped at each phylogenetic level. OTUs with total counts <10 in fewer than 2 samples were filtered out. All of the above steps, except the FastQC reads quality check, were performed with the QIIME 1.9 open-source bioinformatics pipeline for microbiome analysis [55]. The command lines and parameters used to process 16S rRNA-gene sequence data are detailed in Biscarini et al. 2018, S1 Appendix [56].

### Alpha and beta diversity

The gut microbial diversity was assessed within- (alpha diversity) and across- (beta diversity) samples. All indices (alpha and beta diversity) were estimated from the complete OTU table (at the OTU level), filtered for OTUs with more than 10 total counts distributed in at least two samples and normalized for uneven sequencing depth by cumulative sum scaling (CSS, [57]). Besides the number of observed OTUs directly counted from the OTU table, within-sample microbial richness and diversity were estimated using the following indices: Chao1 and ACE (Abundance-based coverage Estimator) for richness, Shannon, Simpson and Fisher’s alpha for diversity [58–63], Simpson E and Pielou’s J (Shannon’s evenness) for evenness [64]. The across-sample gut microbiota diversity was quantified by calculating Bray-Curtis dissimilarities [65]. Among groups (traditional, oil and white diets) and pairwise Bray-Curtis dissimilarities were evaluated non-parametrically using the permutational analysis of variance approach (999 permutations; [66]). Details on the calculation of the mentioned alpha- and beta-diversity indices can be found in Biscarini et al. (S2 Appendix, [56]).

### Statistical analysis

The gut microbiome has been sampled at four consecutive timepoints and measurements of the microbiome were therefore repeated over time. Hence, observations could not be assumed to be independent from each other, but were correlated within individual pigs and across time. This was taken into account in the linear models used to analyse between-group (treatments, timepoints) differences in terms of alpha diversity indices and OTU counts:

$$\begin{array}{c} y_{\text{ijk}}=\mu +pig_{j}+{\left[treatment|timepoint\right]}_{k(j)}+e_{ijk} \end{array}$$ (1)

Where *y*_*ijk*_ is the alpha diversity index value or OTU counts for record *i* from pig *j* with treatment or timepoint *k*; μ is the intercept, *pig*_*j*_ is the random effect of the individual pig, *treatment*|*timepoint*_*k*(*j*)_ is the systematic effect of treatment or timepoint *k* nested within pig *j*, and *e*_*ijk*_ is the residual. $Var\left(y\right)=\Sigma +\text{I}*\sigma_{\text{e}}^{2}$, where **Σ** is a block diagonal matrix, with 1s on the diagonal and the covariances *σ*_*i*,*j*_ between records within pigs in the off-diagonal block elements; **I** is the identity matrix and $\sigma_{e}^{2}$ is the residual variance. First-order autocorrelations were used to model covariation of pig measurements between timepoints. For the F:B ratio, the interaction between treatments and timepoints (pre-/post-weaning) was tested by expanding model 1 as follows:

$$\begin{array}{c} y_{\text{ijk}}=\mu +pig_{j}+treatment_{k(j)}+timepoint_{z(j)}+{\left(treatment*timepoint\right)}_{kz(j)}+e_{ijkz} \end{array}$$ (2)

Where terms were as in model 1 with the addition of the interaction terms treatment*timepoint. Besides correctly accounting for not independent nested observations, multilevel models as those in Eqs 1 and 2 have the property of increasing the power of analysis through lower between-subject variability (each subject is its own control, fewer degrees of freedom).

The F:B ratio was analysed also using bootstrapping: 1000 replicates of the data were resampled with replacement from the original data. The individual F:B ratio was recalculated for each sample in each replicate, and model 1 was fitted to each replicate of the data.

## Supporting information

S1 Fig Rarefaction curves. Sequence-based (left) and sample-based (right) rarefaction curves for the sampled gut microbiotas. Number of detected OTUs on the y-axis; number of sequences (left) and of samples (right) on the x-axis.
(PDF)

S1 Table Sequencing metrics. Average number of 16S rRNA-gene sequences (+/- standard deviation) per experimental group and timepoint.
(PDF)

S2 Table Phylum relative distribution. Relative abundances of phyla in the piglet gut microbiota per treatment (diet group) and timepoint.
(PDF)

S3 Table Relative distribution of taxa. Relative abundances of taxonomic levels (class, order, family, genus) in the piglet gut microbiota per treatment (diet group) and timepoint.
(PDF)

S4 Table OTU significant differences. OTUs significantly different between treatments before and after weaning.
(PDF)
