# Correction: Gut microbiome modifications over time when removing in-feed antibiotics from the prophylaxis of post-weaning diarrhea in piglets

**DOI:** 10.1371/journal.pone.0316120

**Published:** 2024-12-17

**Authors:** Paola Cremonesi, Filippo Biscarini, Bianca Castiglioni, Carlo Angelo Sgoifo, Riccardo Compiani, Paolo Moroni

An additional affiliation is missing for the sixth author. Paolo Moroni is also affiliated with the Quality Milk Production Services, Animal Health Diagnostic Center, Cornell University, Ithaca, NY 14853, United States of America.

## References

[1] Cremonesi P, Biscarini F, Castiglioni B, Sgoifo CA, Compiani R, Moroni P (2022) Gut microbiome modifications over time when removing in-feed antibiotics from the prophylaxis of post-weaning diarrhea in piglets. PLOS ONE 17(3): e0262199. doi: 10.1371/journal.pone.0262199 35255081 PMC8901073

