# Supplementary material for: Gut microbiome modifications over time when removing in-feed antibiotics from the prophylaxis of post-weaning diarrhea in piglets
Source: PLoS One. 2022 Mar 7;17(3):e0262199. doi: 10.1371/journal.pone.0262199 (PMC8901073; doi:10.1371/journal.pone.0262199)
Supplement: S1 Fig — Sequence-based (left) and sample-based (right) rarefaction curves for the sampled gut microbiotas. Number of detected OTUs on the y-axis; number of sequences (left) and of samples (right) on the x-axis. (PDF) [file pone.0262199.s001.pdf]

Gut microbiome modifications over time when removing in-feed antibiotics from the prophylaxis of post-weaning diarrhea in piglets

Paola Cremonesi[1☯] et al.*,

1 National Research Council, Institute of Biology and Biotechnology in Agriculture (CNR-IBBA), Milan, Italy
☯Paola Cremonesi and Filippo Biscarini contributed equally to this work.
*E-mail: filippo.biscarini@ibba.cnr.it

Supplementary Material

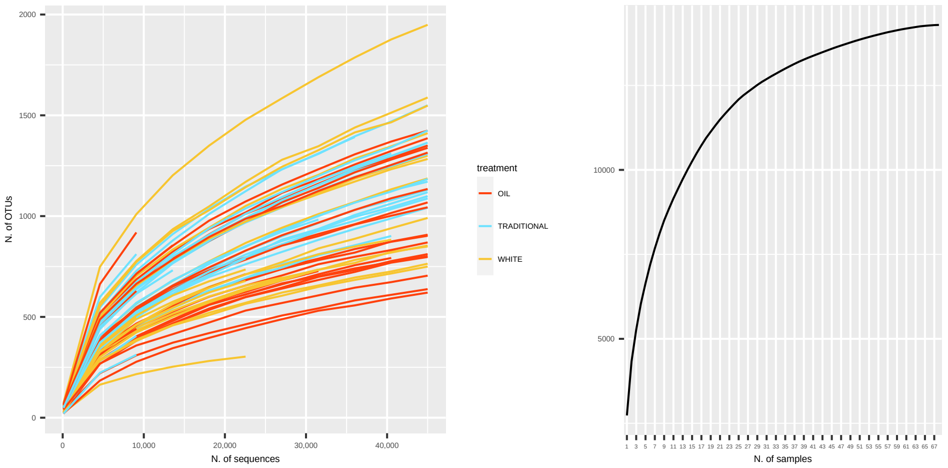

S1 Figure 1. Rarefaction curves. Sequence-based (left) and sample-based (right) rarefaction curves for the sampled gut microbiotas. Number of detected OTUs on the y-axis; number of sequences (left) and of samples (right) on the x-axis.
