# Supplementary material for: Gut microbiome modifications over time when removing in-feed antibiotics from the prophylaxis of post-weaning diarrhea in piglets
Source: PLoS One. 2022 Mar 7;17(3):e0262199. doi: 10.1371/journal.pone.0262199 (PMC8901073; doi:10.1371/journal.pone.0262199)
Supplement: S1 Table — Average number of 16S rRNA-gene sequences (+/- standard deviation) per experimental group and timepoint. (PDF) [file pone.0262199.s002.pdf]

Gut microbiome modifications over time when removing in-feed antibiotics from the prophylaxis of post-weaning diarrhea in piglets

Paola Cremonesi[1◕] et al.*,

1 National Research Council, Institute of Biology and Biotechnology in Agriculture (CNR-IBBA), Milan, Italy
◕Paola Cremonesi and Filippo Biscarini contributed equally to this work.
*E-mail: filippo.biscarini@ibba.cnr.it

Supplementary Material

S1 Table 1. Average number of 16S rRNA-gene sequences (+/- standard deviation) per experimental group and timepoint

treatment	T0	T1	T2	T3
oil	55367.2+/-21348.7	81026.8+/-51291.1	69871.3+/-59729.7	110015.2+/-37496.9
traditional	46274+/-31807.1	40654.8+/-26176.7	99087.8+/-77029.3	143167+/-61104.9
white	27621.4+/-11270.9	97793.8+/-107045.6	110918.4+/-30251.9	153425+/-97899.7
