# Supplementary material for: Gut microbiome modifications over time when removing in-feed antibiotics from the prophylaxis of post-weaning diarrhea in piglets
Source: PLoS One. 2022 Mar 7;17(3):e0262199. doi: 10.1371/journal.pone.0262199 (PMC8901073; doi:10.1371/journal.pone.0262199)
Supplement: S2 Table — Relative abundances of phyla in the piglet gut microbiota per treatment (diet group) and timepoint. (PDF) [file pone.0262199.s003.pdf]

Gut microbiome modifications over time when removing in-feed antibiotics from the prophylaxis of post-weaning diarrhea in piglets

Paola Cremonesi[1◑] et al.*,

1 National Research Council, Institute of Biology and Biotechnology in Agriculture (CNR-IBBA), Milan, Italy
◑Paola Cremonesi and Filippo Biscarini contributed equally to this work.
*E-mail: filippo.biscarini@ibba.cnr.it

Supplementary Material

Table S2. Relative abundances of phyla in the piglet gut microbiota per treatment (diet group) and timepoint

OTU	Group	T0	T1	T2	T3
Firmicutes	OIL	0.3457007	0.4965781	0.6117502	0.5527304
Firmicutes	TRADITIONAL	0.4781912	0.6000563	0.5184750	0.5933683
Firmicutes	WHITE	0.3999341	0.4909771	0.5766113	0.5489473
Proteobacteria	OIL	0.3608533	0.1075891	0.0816246	0.0643048
Proteobacteria	TRADITIONAL	0.2305087	0.1245515	0.1340380	0.0573324
Proteobacteria	WHITE	0.4234811	0.0995669	0.0679742	0.0643441
Bacteroidetes	OIL	0.1881828	0.3237184	0.1991300	0.3382592
Bacteroidetes	TRADITIONAL	0.2200051	0.1849144	0.2623070	0.3047097
Bacteroidetes	WHITE	0.1089285	0.3338071	0.2879556	0.3388248
Actinobacteria	OIL	0.0483121	0.0056372	0.0296781	0.0144163
Actinobacteria	TRADITIONAL	0.0387778	0.0506774	0.0297281	0.0167669
Actinobacteria	WHITE	0.0411479	0.0109545	0.0148328	0.0138426
Fusobacteria	OIL	0.0530341	0.0477254	0.0134282	0.0023793
Fusobacteria	TRADITIONAL	0.0264944	0.0130531	0.0208698	0.0015003
Fusobacteria	WHITE	0.0082892	0.0360510	0.0105268	0.0015393
Spirochaetae	OIL	0.0009475	0.0025469	0.0152098	0.0091067
Spirochaetae	TRADITIONAL	0.0006836	0.0050867	0.0052183	0.0076053
Spirochaetae	WHITE	0.0052676	0.0063390	0.0106178	0.0080722
Cyanobacteria	OIL	0.0007371	0.0003559	0.0032601	0.0085698
Cyanobacteria	TRADITIONAL	0.0015142	0.0014556	0.0026637	0.0112297
Cyanobacteria	WHITE	0.0026572	0.0039078	0.0076300	0.0106835
Lentisphaerae	OIL	0.0007167	0.0047319	0.0134218	0.0017623
Lentisphaerae	TRADITIONAL	0.0020360	0.0034320	0.0048918	0.0000668
Lentisphaerae	WHITE	0.0017838	0.0049271	0.0071591	0.0020858
Euryarchaeota	OIL	0.0000000	0.0002693	0.0094821	0.0015976
Euryarchaeota	TRADITIONAL	0.0000000	0.0062851	0.0035778	0.0012343
Euryarchaeota	WHITE	0.0039005	0.0016517	0.0031861	0.0025414
Tenericutes	OIL	0.0008688	0.0023627	0.0018763	0.0045327
Tenericutes	TRADITIONAL	0.0005559	0.0015937	0.0013215	0.0058496

Tenericutes	WHITE	0.0021168	0.0008294	0.0018812	0.0041426
Synergistetes	OIL	0.0006468	0.0070049	0.0156025	0.0010191
Synergistetes	TRADITIONAL	0.0012331	0.0067415	0.0134796	0.0001414
Synergistetes	WHITE	0.0009798	0.0092050	0.0071518	0.0010015
Planctomycetes	OIL	0.0000000	0.0002227	0.0038124	0.0005162
Planctomycetes	TRADITIONAL	0.0000000	0.0021527	0.0026630	0.0000000
Planctomycetes	WHITE	0.0011881	0.0009796	0.0015531	0.0006012
Elusimicrobia	OIL	0.0000000	0.0012574	0.0017239	0.0008056
Elusimicrobia	TRADITIONAL	0.0000000	0.0000000	0.0007665	0.0001953
Elusimicrobia	WHITE	0.0003253	0.0008040	0.0029202	0.0033736
