# Supplementary material for: Gut microbiome modifications over time when removing in-feed antibiotics from the prophylaxis of post-weaning diarrhea in piglets
Source: PLoS One. 2022 Mar 7;17(3):e0262199. doi: 10.1371/journal.pone.0262199 (PMC8901073; doi:10.1371/journal.pone.0262199)
Supplement: S3 Table — Relative abundances of taxonomic levels (class, order, family, genus) in the piglet gut microbiota per treatment (diet group) and timepoint. (PDF) [file pone.0262199.s004.pdf]

Gut microbiome modifications over time when removing in-feed antibiotics from the prophylaxis of post-weaning diarrhea in piglets

Paola Cremonesi[1◑] et al.*,

1 National Research Council, Institute of Biology and Biotechnology in Agriculture (CNR-IBBA), Milan, Italy
◑Paola Cremonesi and Filippo Biscarini contributed equally to this work.
*E-mail: filippo.biscarini@ibba.cnr.it

Supplementary Material

S3 Table. Relative abundances of taxonomic levels (class, order, family, genus) in the piglet gut microbiota per treatment (diet group) and timepoint

level	OTU	Group	T0	T1	T2	T3
class	Actinobacteria	OIL	0.0328361	0.0023231	0.0130981	0.0041072
class	Actinobacteria	TRADITIONAL	0.0226294	0.0341353	0.0165874	0.0048349
class	Actinobacteria	WHITE	0.0315033	0.0031762	0.0057967	0.0020594
class	Bacilli	OIL	0.1076515	0.0638553	0.0454990	0.0450303
class	Bacilli	TRADITIONAL	0.0904687	0.0822586	0.0630952	0.0510976
class	Bacilli	WHITE	0.1261738	0.0589740	0.0456077	0.0314513
class	Bacteroidia	OIL	0.1785307	0.3323483	0.2120726	0.3496514
class	Bacteroidia	TRADITIONAL	0.2209792	0.1847438	0.2728815	0.3119688
class	Bacteroidia	WHITE	0.0930576	0.3465481	0.3025608	0.3521925
class	Betaproteobacteria	OIL	0.0165464	0.0202716	0.0093557	0.0059228
class	Betaproteobacteria	TRADITIONAL	0.0047951	0.0091201	0.0154085	0.0093440
class	Betaproteobacteria	WHITE	0.0158041	0.0146578	0.0148841	0.0065197
class	Clostridia	OIL	0.1764550	0.3406684	0.5511287	0.4037439
class	Clostridia	TRADITIONAL	0.3331573	0.4875560	0.4040586	0.4608689
class	Clostridia	WHITE	0.2390117	0.3625813	0.4901629	0.4061235
class	Coriobacteriia	OIL	0.0172607	0.0034938	0.0190808	0.0108725
class	Coriobacteriia	TRADITIONAL	0.0171635	0.0192430	0.0148610	0.0124656
class	Coriobacteriia	WHITE	0.0120700	0.0082849	0.0099526	0.0123957
class	Epsilonproteobacteria	OIL	0.0124691	0.0138630	0.0188370	0.0234543
class	Epsilonproteobacteria	TRADITIONAL	0.0204606	0.0192547	0.0285064	0.0130317
class	Epsilonproteobacteria	WHITE	0.0068308	0.0315069	0.0163495	0.0121459
class	Erysipelotrichia	OIL	0.0153744	0.0129767	0.0180424	0.0259360
class	Erysipelotrichia	TRADITIONAL	0.0151635	0.0200713	0.0159902	0.0197035
class	Erysipelotrichia	WHITE	0.0152936	0.0138160	0.0129970	0.0237786
class	Fusobacteriia	OIL	0.0549933	0.0492466	0.0145598	0.0024723
class	Fusobacteriia	TRADITIONAL	0.0271880	0.0137487	0.0220774	0.0015481
class	Fusobacteriia	WHITE	0.0087778	0.0377182	0.0111772	0.0016074
class	Gammaproteobacteria	OIL	0.3287344	0.0658958	0.0477597	0.0279802
class	Gammaproteobacteria	TRADITIONAL	0.1959507	0.0877182	0.0805996	0.0335740

class	Gammaproteobacteria	WHITE	0.4084487	0.0442366	0.0262717	0.0386671
class	Negativicutes	OIL	0.0589904	0.0949049	0.0486288	0.0996211
class	Negativicutes	TRADITIONAL	0.0519207	0.0421502	0.0653333	0.0805835
class	Negativicutes	WHITE	0.0430286	0.0783120	0.0634715	0.1118821
order	Bacteroidales	OIL	0.2014584	0.3375605	0.2170461	0.3590440
order	Bacteroidales	TRADITIONAL	0.2324335	0.1957191	0.2824942	0.3182333
order	Bacteroidales	WHITE	0.1089191	0.3538678	0.3084888	0.3614145
order	Burkholderiales	OIL	0.0150974	0.0196209	0.0086183	0.0059404
order	Burkholderiales	TRADITIONAL	0.0040391	0.0089702	0.0147176	0.0090523
order	Burkholderiales	WHITE	0.0163209	0.0147435	0.0140547	0.0065970
order	Campylobacterales	OIL	0.0140704	0.0140804	0.0192787	0.0240843
order	Campylobacterales	TRADITIONAL	0.0215211	0.0203986	0.0295106	0.0132934
order	Campylobacterales	WHITE	0.0079951	0.0321724	0.0166698	0.0124639
order	Clostridiales	OIL	0.1991162	0.3460111	0.5640537	0.4145896
order	Clostridiales	TRADITIONAL	0.3504263	0.5165207	0.4182922	0.4701234
order	Clostridiales	WHITE	0.2797509	0.3702396	0.4997666	0.4167577
order	Coriobacteriales	OIL	0.0194774	0.0035486	0.0195283	0.0111646
order	Coriobacteriales	TRADITIONAL	0.0180532	0.0203862	0.0153845	0.0127160
order	Coriobacteriales	WHITE	0.0141273	0.0084599	0.0101476	0.0127203
order	Enterobacteriales	OIL	0.2740552	0.0325504	0.0285704	0.0047373
order	Enterobacteriales	TRADITIONAL	0.1697565	0.0696352	0.0562080	0.0166199
order	Enterobacteriales	WHITE	0.3316343	0.0114754	0.0076727	0.0104791
order	Erysipelotrichales	OIL	0.0173488	0.0131802	0.0184656	0.0266327
order	Erysipelotrichales	TRADITIONAL	0.0159494	0.0212637	0.0165535	0.0200992
order	Erysipelotrichales	WHITE	0.0179004	0.0141078	0.0132517	0.0244012
order	Fusobacteriales	OIL	0.0620558	0.0500189	0.0149012	0.0025387
order	Fusobacteriales	TRADITIONAL	0.0285973	0.0145655	0.0228552	0.0015792
order	Fusobacteriales	WHITE	0.0102739	0.0385149	0.0113962	0.0016495
order	Lactobacillales	OIL	0.0968672	0.0630188	0.0412996	0.0440663
order	Lactobacillales	TRADITIONAL	0.0867112	0.0754674	0.0588442	0.0488758
order	Lactobacillales	WHITE	0.1074809	0.0567322	0.0422274	0.0304764
order	Pasteurellales	OIL	0.0326536	0.0227720	0.0101449	0.0033578
order	Pasteurellales	TRADITIONAL	0.0145829	0.0067273	0.0121511	0.0060589
order	Pasteurellales	WHITE	0.0537169	0.0163780	0.0077178	0.0067185
order	Selenomonadales	OIL	0.0665663	0.0963933	0.0497693	0.1022972
order	Selenomonadales	TRADITIONAL	0.0546119	0.0446543	0.0676347	0.0822016
order	Selenomonadales	WHITE	0.0503627	0.0799661	0.0647151	0.1148117
family	Acidaminococcaceae	OIL	0.0262223	0.0438282	0.0279671	0.0247565
family	Acidaminococcaceae	TRADITIONAL	0.0228512	0.0296590	0.0334536	0.0299558
family	Acidaminococcaceae	WHITE	0.0153070	0.0372932	0.0285698	0.0313233
family	Bacteroidaceae	OIL	0.1380044	0.0532346	0.0513782	0.0096353
family	Bacteroidaceae	TRADITIONAL	0.1171100	0.0755544	0.0821112	0.0076364
family	Bacteroidaceae	WHITE	0.0217453	0.0780370	0.0291851	0.0082274
family	Bacteroidales S24-7 group	OIL	0.0064988	0.0392185	0.0263543	0.0373217
family	Bacteroidales S24-7 group	TRADITIONAL	0.0203441	0.0130599	0.0301409	0.0272994
family	Bacteroidales S24-7 group	WHITE	0.0184228	0.0269616	0.0415924	0.0444582
family	Christensenellaceae	OIL	0.0024749	0.0069854	0.0631851	0.0096181
family	Christensenellaceae	TRADITIONAL	0.0105403	0.0547403	0.0342412	0.0027404
family	Christensenellaceae	WHITE	0.0202560	0.0089354	0.0256313	0.0126361
family	Clostridiaceae 1	OIL	0.0439347	0.0037043	0.0155107	0.0090509

family	Clostridiaceae 1	TRADITIONAL	0.0410246	0.0280880	0.0181278	0.0090095
family	Clostridiaceae 1	WHITE	0.0181685	0.0090040	0.0048339	0.0108903
family	Coriobacteriaceae	OIL	0.0215694	0.0037984	0.0211363	0.0115589
family	Coriobacteriaceae	TRADITIONAL	0.0197340	0.0219947	0.0166267	0.0130625
family	Coriobacteriaceae	WHITE	0.0152041	0.0092074	0.0106971	0.0129632
family	Enterobacteriaceae	OIL	0.3034904	0.0348412	0.0309229	0.0049046
family	Enterobacteriaceae	TRADITIONAL	0.1855615	0.0751296	0.0607464	0.0170729
family	Enterobacteriaceae	WHITE	0.3569116	0.0124893	0.0080881	0.0106793
family	Erysipelotrichaceae	OIL	0.0192122	0.0141078	0.0199860	0.0275735
family	Erysipelotrichaceae	TRADITIONAL	0.0174344	0.0229414	0.0178901	0.0206470
family	Erysipelotrichaceae	WHITE	0.0192647	0.0153543	0.0139692	0.0248673
family	Family XIII	OIL	0.0025370	0.0118839	0.0181460	0.0124680
family	Family XIII	TRADITIONAL	0.0068461	0.0138143	0.0144144	0.0091769
family	Family XIII	WHITE	0.0128671	0.0110615	0.0178216	0.0092123
family	Fusobacteriaceae	OIL	0.0388841	0.0308548	0.0110211	0.0015872
family	Fusobacteriaceae	TRADITIONAL	0.0196085	0.0100814	0.0145789	0.0009415
family	Fusobacteriaceae	WHITE	0.0054873	0.0247967	0.0074142	0.0009556
family	Lachnospiraceae	OIL	0.1024561	0.1567410	0.1849989	0.1568650
family	Lachnospiraceae	TRADITIONAL	0.1779105	0.2188820	0.1786714	0.1777508
family	Lachnospiraceae	WHITE	0.0988405	0.1955990	0.2281095	0.1532517
family	Lactobacillaceae	OIL	0.0338827	0.0596697	0.0224183	0.0384835
family	Lactobacillaceae	TRADITIONAL	0.0414368	0.0424678	0.0352269	0.0403540
family	Lactobacillaceae	WHITE	0.0421764	0.0451820	0.0327779	0.0252822
family	Pasteurellaceae	OIL	0.0361608	0.0243746	0.0109802	0.0034764
family	Pasteurellaceae	TRADITIONAL	0.0159406	0.0072581	0.0131322	0.0062241
family	Pasteurellaceae	WHITE	0.0578113	0.0178250	0.0081357	0.0068468
family	Porphyromonadaceae	OIL	0.0479423	0.0406378	0.0361208	0.0099243
family	Porphyromonadaceae	TRADITIONAL	0.0489367	0.0580596	0.0519983	0.0101032
family	Porphyromonadaceae	WHITE	0.0113472	0.0457411	0.0331068	0.0106740
family	Prevotellaceae	OIL	0.0266624	0.2026728	0.0836262	0.2902005
family	Prevotellaceae	TRADITIONAL	0.0449762	0.0281538	0.1140188	0.2667601
family	Prevotellaceae	WHITE	0.0517812	0.2086538	0.1930303	0.2789892
family	Rikenellaceae	OIL	0.0037888	0.0214271	0.0299050	0.0193177
family	Rikenellaceae	TRADITIONAL	0.0222924	0.0354518	0.0242841	0.0107626
family	Rikenellaceae	WHITE	0.0098460	0.0220828	0.0239054	0.0215193
family	Ruminococcaceae	OIL	0.0405008	0.1781685	0.2905015	0.2303390
family	Ruminococcaceae	TRADITIONAL	0.1067064	0.2120460	0.1847388	0.2709194
family	Ruminococcaceae	WHITE	0.1222816	0.1601894	0.2322537	0.2278792
family	Streptococcaceae	OIL	0.0343389	0.0055598	0.0149309	0.0053567
family	Streptococcaceae	TRADITIONAL	0.0259099	0.0209958	0.0221507	0.0057483
family	Streptococcaceae	WHITE	0.0331112	0.0104602	0.0078060	0.0048587
family	Veillonellaceae	OIL	0.0474936	0.0593492	0.0259002	0.0811543
family	Veillonellaceae	TRADITIONAL	0.0368453	0.0185186	0.0396422	0.0544861
family	Veillonellaceae	WHITE	0.0388944	0.0497383	0.0396493	0.0856815
genus	[Eubacterium] coprostanoligenes group	OIL	0.0057538	0.0206322	0.0411418	0.0282824
genus	[Eubacterium] coprostanoligenes group	TRADITIONAL	0.0192088	0.0337223	0.0295013	0.0290619
genus	[Eubacterium] coprostanoligenes group	WHITE	0.0220385	0.0202686	0.0529907	0.0349453
genus	Alloprevotella	OIL	0.0088581	0.0286417	0.0156199	0.0350291
genus	Alloprevotella	TRADITIONAL	0.0141675	0.0086413	0.0165580	0.0340825
genus	Alloprevotella	WHITE	0.0111386	0.0295940	0.0282983	0.0311968

genus	Bacteroides	OIL	0.1869325	0.0744185	0.0756928	0.0142244
genus	Bacteroides	TRADITIONAL	0.1623057	0.1109296	0.1158739	0.0108971
genus	Bacteroides	WHITE	0.0321749	0.1121814	0.0446945	0.0121033
genus	Blautia	OIL	0.0101330	0.0157365	0.0327980	0.0213935
genus	Blautia	TRADITIONAL	0.0317614	0.0286738	0.0252629	0.0302174
genus	Blautia	WHITE	0.0141309	0.0148374	0.0190350	0.0180473
genus	Christensenellaceae R-7 group	OIL	0.0033524	0.0097651	0.0919750	0.0135345
genus	Christensenellaceae R-7 group	TRADITIONAL	0.0140300	0.0785273	0.0468958	0.0038079
genus	Christensenellaceae R-7 group	WHITE	0.0296367	0.0125343	0.0390529	0.0178354
genus	Clostridium sensu stricto 1	OIL	0.0497572	0.0038079	0.0191741	0.0105913
genus	Clostridium sensu stricto 1	TRADITIONAL	0.0438012	0.0362876	0.0221382	0.0098594
genus	Clostridium sensu stricto 1	WHITE	0.0236039	0.0102629	0.0036814	0.0129417
genus	Escherichia-Shigella	OIL	0.3130406	0.0393808	0.0364677	0.0056628
genus	Escherichia-Shigella	TRADITIONAL	0.1944597	0.0831282	0.0685755	0.0196678
genus	Escherichia-Shigella	WHITE	0.4311491	0.0137473	0.0096131	0.0120179
genus	Faecalibacterium	OIL	0.0010094	0.0314292	0.0077423	0.0504332
genus	Faecalibacterium	TRADITIONAL	0.0064970	0.0053787	0.0072649	0.1141794
genus	Faecalibacterium	WHITE	0.0090240	0.0266048	0.0242205	0.0508516
genus	Fusobacterium	OIL	0.0515960	0.0423931	0.0162368	0.0023431
genus	Fusobacterium	TRADITIONAL	0.0271759	0.0148015	0.0204074	0.0013435
genus	Fusobacterium	WHITE	0.0078170	0.0350721	0.0112322	0.0014058
genus	Lachnoclostridium	OIL	0.0301203	0.0300900	0.0367139	0.0157710
genus	Lachnoclostridium	TRADITIONAL	0.0642936	0.0580594	0.0462092	0.0181289
genus	Lachnoclostridium	WHITE	0.0068661	0.0394057	0.0315969	0.0162599
genus	Lachnospiraceae NK4A136 group	OIL	0.0044424	0.0134203	0.0265051	0.0150275
genus	Lachnospiraceae NK4A136 group	TRADITIONAL	0.0106720	0.0303072	0.0205796	0.0181855
genus	Lachnospiraceae NK4A136 group	WHITE	0.0160856	0.0232363	0.0367186	0.0180144
genus	Lactobacillus	OIL	0.0455650	0.0834144	0.0324027	0.0559988
genus	Lactobacillus	TRADITIONAL	0.0568207	0.0614458	0.0489705	0.0561342
genus	Lactobacillus	WHITE	0.0611738	0.0646578	0.0492128	0.0369605
genus	Megasphaera	OIL	0.0198835	0.0146209	0.0145067	0.0283687
genus	Megasphaera	TRADITIONAL	0.0107288	0.0066400	0.0152888	0.0167443
genus	Megasphaera	WHITE	0.0101274	0.0188712	0.0169414	0.0252635
genus	Parabacteroides	OIL	0.0328732	0.0325503	0.0324539	0.0136316
genus	Parabacteroides	TRADITIONAL	0.0348752	0.0539920	0.0402547	0.0132784
genus	Parabacteroides	WHITE	0.0114168	0.0370978	0.0373620	0.0145326
genus	Phascolarctobacterium	OIL	0.0260280	0.0571185	0.0366093	0.0300374
genus	Phascolarctobacterium	TRADITIONAL	0.0287561	0.0418296	0.0434741	0.0361187
genus	Phascolarctobacterium	WHITE	0.0184501	0.0489562	0.0367322	0.0375033
genus	Prevotella 1	OIL	0.0011130	0.0156001	0.0094529	0.0209053
genus	Prevotella 1	TRADITIONAL	0.0021212	0.0015697	0.0184758	0.0362448
genus	Prevotella 1	WHITE	0.0051651	0.0247830	0.0336293	0.0297362
genus	Prevotella 2	OIL	0.0029789	0.1005112	0.0346972	0.0596027
genus	Prevotella 2	TRADITIONAL	0.0158437	0.0101039	0.0258002	0.0456622
genus	Prevotella 2	WHITE	0.0137778	0.0772362	0.0586866	0.0476773
genus	Prevotella 7	OIL	0.0030692	0.0206659	0.0100683	0.0429665
genus	Prevotella 7	TRADITIONAL	0.0031203	0.0031179	0.0105027	0.0268080
genus	Prevotella 7	WHITE	0.0071902	0.0192538	0.0148771	0.0407536
genus	Prevotella 9	OIL	0.0107175	0.0606036	0.0189009	0.1696287
genus	Prevotella 9	TRADITIONAL	0.0077760	0.0078026	0.0448089	0.1584636

genus	Prevotella 9	WHITE	0.0142788	0.0785946	0.0721412	0.1580341
genus	Prevotellaceae NK3B31 group	OIL	0.0021846	0.0185410	0.0121374	0.0337821
genus	Prevotellaceae NK3B31 group	TRADITIONAL	0.0135805	0.0038584	0.0242617	0.0333039
genus	Prevotellaceae NK3B31 group	WHITE	0.0107084	0.0299623	0.0389192	0.0392521
genus	Rikenellaceae RC9 gut group	OIL	0.0018987	0.0170154	0.0243288	0.0259902
genus	Rikenellaceae RC9 gut group	TRADITIONAL	0.0149248	0.0154815	0.0130422	0.0148875
genus	Rikenellaceae RC9 gut group	WHITE	0.0116584	0.0146101	0.0210457	0.0296800
genus	Ruminiclostridium 9	OIL	0.0034419	0.0229819	0.0300297	0.0152573
genus	Ruminiclostridium 9	TRADITIONAL	0.0089605	0.0230013	0.0182085	0.0211936
genus	Ruminiclostridium 9	WHITE	0.0047206	0.0216331	0.0203844	0.0153694
genus	Ruminococcaceae UCG-002	OIL	0.0021210	0.0249107	0.0475921	0.0188942
genus	Ruminococcaceae UCG-002	TRADITIONAL	0.0191028	0.0424753	0.0305456	0.0057140
genus	Ruminococcaceae UCG-002	WHITE	0.0190076	0.0103780	0.0174045	0.0209941
genus	Ruminococcaceae UCG-005	OIL	0.0020813	0.0082372	0.0354466	0.0278833
genus	Ruminococcaceae UCG-005	TRADITIONAL	0.0056215	0.0273926	0.0222159	0.0189760
genus	Ruminococcaceae UCG-005	WHITE	0.0116568	0.0058658	0.0138263	0.0269976
genus	Ruminococcaceae UCG-014	OIL	0.0005568	0.0216862	0.0339900	0.0385694
genus	Ruminococcaceae UCG-014	TRADITIONAL	0.0008819	0.0026613	0.0091120	0.0539904
genus	Ruminococcaceae UCG-014	WHITE	0.0149211	0.0146576	0.0223865	0.0368309
genus	Streptococcus	OIL	0.0465135	0.0075890	0.0214326	0.0068798
genus	Streptococcus	TRADITIONAL	0.0359091	0.0301914	0.0305630	0.0075402
genus	Streptococcus	WHITE	0.0486902	0.0148783	0.0111496	0.0066060
