# Supplementary material for: Gut microbiome modifications over time when removing in-feed antibiotics from the prophylaxis of post-weaning diarrhea in piglets
Source: PLoS One. 2022 Mar 7;17(3):e0262199. doi: 10.1371/journal.pone.0262199 (PMC8901073; doi:10.1371/journal.pone.0262199)
Supplement: S4 Table — OTUs significantly different between treatments before and after weaning. (PDF) [file pone.0262199.s005.pdf]

Gut microbiome modifications over time when removing in-feed antibiotics from the prophylaxis of post-weaning diarrhea in piglets

Paola Cremonesi[1◑] et al.*,

1 National Research Council, Institute of Biology and Biotechnology in Agriculture (CNR-IBBA), Milan, Italy
◑Paola Cremonesi and Filippo Biscarini contributed equally to this work.
*E-mail: filippo.biscarini@ibba.cnr.it

Supplementary Material

S4 Table. OTUs significantly different between treatments before and after weaning (TR_pre, TR_post: traditional diet group, pre and post weaning; WH_pre, WH_post: white diet group, pre and post weaning)

taxa	OTU	p.value	OIL_pre	OIL_post	TR_pre	TR_post	WH_pre	WH_post
phylum	Actinobacteria	0.0151	116.28	133.29	211.39	150.43	106.89	106.81
phylum	Elusimicrobia	0.0084	3.26	7.64	0.00	3.03	2.85	23.32
phylum	Fusobacteria	0.0371	235.19	46.44	91.15	68.69	116.27	46.57
class	Actinobacteria	0.0162	72.48	47.84	129.08	64.40	63.61	28.30
class	Bacteroidetes VC2.1 Bac22	0.0026	2.69	4.75	17.45	9.82	4.00	3.38
class	Elusimicrobia	0.0084	3.26	7.64	0.00	3.03	2.85	23.32
class	Fusobacteriia	0.0371	235.19	46.44	91.15	68.69	116.27	46.57
order	Actinomycetales	0.0118	12.10	15.45	38.38	11.04	3.94	0.22
order	Aeromonadales	0.0007	29.65	56.62	7.01	26.62	32.18	82.53
order	Elusimicrobiales	0.0084	3.26	7.64	0.00	3.03	2.85	23.32
order	Fusobacteriales	0.0371	235.19	46.44	91.15	68.69	116.27	46.57
order	Rhodospirillales	0.0002	0.78	4.05	0.00	1.25	2.58	14.52
order	uncultured rumen bacterium	0.0155	0.86	4.60	0.84	1.11	0.95	11.41
family	Actinomycetaceae	0.0118	12.10	15.45	38.38	11.04	3.94	0.22
family	CFT112H7	0.0269	78.00	12.02	26.07	20.06	32.20	15.29
family	Clostridiaceae 1	0.0138	79.91	64.43	134.94	76.41	46.91	51.74
family	Elusimicrobiaceae	0.0084	3.26	7.64	0.00	3.03	2.85	23.32
family	Fusobacteriaceae	0.0435	134.37	31.10	57.63	40.49	67.31	28.48
family	Peptostreptococcaceae	0.0080	42.35	35.77	74.30	42.92	41.38	29.11
family	Prevotellaceae	0.0486	511.06	1078.80	142.52	1178.95	574.21	1562.38
family	Pseudomonadaceae	0.0375	12.22	12.83	6.26	9.20	20.54	13.09
family	Succinivibrionaceae	0.0005	29.23	56.62	5.46	26.62	32.18	82.53
family	uncultured rumen bacterium	0.0266	0.29	1.97	0.28	0.93	0.55	4.24
genus	[Eubacterium] ruminantium group	0.0037	1.54	13.02	0.19	8.25	7.51	24.10
genus	Actinobacillus	0.0136	55.85	10.05	20.67	17.73	78.72	24.42
genus	Actinomyces	0.0253	8.40	11.97	28.02	6.42	3.94	0.00
genus	Alistipes	0.0115	15.00	16.98	54.99	29.01	20.08	19.61
genus	Anaerobiospirillum	0.0022	21.80	6.24	1.49	3.25	17.29	17.39
genus	Anaerovibrio	0.0058	27.90	27.24	4.31	22.14	24.26	40.73
genus	Blautia	0.0040	38.13	97.02	83.25	116.27	38.11	82.20
genus	Candidatus Soleaferrea	0.0115	1.14	8.57	1.08	4.31	4.35	8.78
genus	Clostridium sensu stricto 1	0.0158	65.95	52.79	110.26	63.17	39.34	36.65
genus	Clostridium sensu stricto 2	0.0456	4.62	0.77	14.07	0.00	0.55	0.26
genus	Collinsella	0.0019	17.50	28.25	39.32	32.59	14.28	19.54

genus	Dialister	0.0268	10.81	63.35	4.06	32.76	18.59	78.26
genus	Eisenbergiella	0.0074	5.57	3.43	9.68	6.88	3.32	1.91
genus	Elusimicrobium	0.0084	3.26	7.64	0.00	3.03	2.85	23.32
genus	Enterococcus	0.0255	39.94	8.12	58.28	5.55	23.37	3.27
genus	Fusobacterium	0.0452	131.86	31.10	57.63	40.19	66.07	28.21
genus	Haemophilus	0.0096	19.26	7.41	7.28	7.46	28.12	10.17
genus	Hungatella	0.0021	2.68	1.21	18.57	4.21	5.77	0.67
genus	Lachnoclostridium	0.0032	85.80	91.82	168.55	126.15	72.43	106.37
genus	Lachnospiraceae FCS020 group	0.0140	4.38	11.58	14.60	18.85	6.57	20.07
genus	Lachnospiraceae ND3007 group	0.0250	1.30	7.06	1.04	12.86	4.99	16.01
genus	Lachnospiraceae NK3A20 group	0.0024	4.33	14.68	6.37	10.78	16.02	19.37
genus	Lachnospiraceae UCG-005	0.0088	6.98	16.62	4.24	12.24	9.69	32.56
genus	Lachnospiraceae UCG-010	0.0064	13.51	7.11	2.10	6.46	20.16	23.57
genus	Megamonas	0.0208	8.93	19.46	10.06	57.23	8.36	17.11
genus	Mitsuokella	0.0001	6.19	22.85	1.32	14.93	13.46	45.28
genus	Odoribacter	0.0466	9.10	1.30	1.35	4.73	13.63	6.92
genus	Oribacterium	0.0001	23.85	38.32	3.66	19.09	23.30	55.88
genus	Parasutterella	0.0247	6.02	2.71	6.17	16.77	0.00	1.32
genus	Pasteurella	0.0068	34.89	18.35	14.13	27.58	9.14	12.99
genus	Peptoclostridium	0.0091	13.90	13.68	31.15	19.94	13.23	15.27
genus	Prevotella 1	0.0234	27.09	58.21	5.08	117.98	46.35	140.51
genus	Prevotella 2	0.0320	169.51	178.52	35.66	153.22	142.27	235.95
genus	Prevotella 7	0.0378	37.80	104.49	8.60	81.52	38.99	122.77
genus	Prevotellaceae NK3B31 group	0.0161	33.23	88.99	23.86	121.64	60.23	173.24
genus	Prevotellaceae UCG-001	0.0002	15.32	41.18	3.17	35.11	19.64	59.19
genus	Prevotellaceae UCG-003	0.0121	7.65	18.82	1.16	18.71	9.10	33.17
genus	Prevotellaceae UCG-004	0.0029	5.17	11.98	2.70	5.05	8.42	17.02
genus	Pseudomonas	0.0243	10.90	12.33	4.96	8.17	17.85	12.67
genus	Ruminococcaceae UCG-004	0.0076	3.24	5.25	9.27	9.43	3.78	7.53
genus	Ruminococcaceae UCG-011	0.0017	9.13	7.61	1.82	4.32	11.75	13.17
genus	Ruminococcus 1	0.0065	10.62	56.67	17.06	37.95	28.75	89.79
genus	Rummeliibacillus	0.0437	3.39	0.00	3.28	0.18	12.36	0.43
genus	Selenomonas	0.0169	0.28	15.31	0.18	7.03	3.11	21.15
genus	Succinivibrio	0.0385	2.34	43.52	3.60	20.63	7.73	53.46
genus	Treponema 2	0.0200	3.08	28.24	2.21	14.20	12.28	28.41
genus	Trueperella	0.0429	3.69	3.48	10.36	4.62	0.00	0.22
